# A Meta‐Analysis of International Flunixin Pharmacokinetics in Horses: Toward Regulatory Harmonization and Individualized Detection Times Using Bayesian Paradigm

**DOI:** 10.1002/dta.3961

**Published:** 2025-10-25

**Authors:** Taisuke Kuroda, Heather K. Knych, Glenys K. Noble, Yohei Minamijima, Gary Ngai‐Wa Leung, Motoi Nomura, Fumiaki Mizobe, Yuhiro Ishikawa, Kanichi Kusano, Pierre‐Louis Toutain

**Affiliations:** ^1^ Clinical Veterinary Medicine Division, Equine Research Institute Japan Racing Association Shimotsuke Japan; ^2^ Graduate School of Agriculture Tokyo University of Agriculture and Technology Fuchu Japan; ^3^ K.L. Maddy Equine Analytical Chemistry Laboratory (Pharmacology Section), School of Veterinary Medicine University of California, Davis California USA; ^4^ Department of Molecular Biosciences, School of Veterinary Medicine University of California, Davis California USA; ^5^ School of Agricultural, Environmental and Veterinary Sciences Charles Sturt University Wagga Wagga New South Wales Australia; ^6^ Drug Analysis Department, Laboratory of Racing Chemistry Utsunomiya Japan; ^7^ Equine Department Main Office Japan Racing Association Tokyo Japan; ^8^ London Representative Office Japan Racing Association London United Kingdom; ^9^ Comparative Biomedical Sciences The Royal Veterinary College London United Kingdom

**Keywords:** flunixin, irrelevant plasma concentration, irrelevant urine concentration, international meta‐analysis, individual Bayesian withdrawal time

## Abstract

Flunixin meglumine is widely used to manage pain and inflammation in horses, and its regulation requires robust pharmacokinetic analysis for harmonization. In this study, we conducted a meta‐analysis of flunixin disposition using plasma and urine concentration data from 65 horses across four countries to robustly estimate pharmacokinetic parameters in setting screening limits (SLs) for controlling medications in horses. A population (POP) model was developed using nonlinear mixed‐effects model analysis. The irrelevant plasma concentration (IPC) and irrelevant urine concentration (IUC) were determined to be 1.9 and 70.2 ng/mL, respectively, with a typical urine‐to‐plasma ratio (Rss) of 35.9. Using the current International Federation of Horseracing Authorities (IFHA) screening limits (ISLs) (1 ng/mL for plasma; 100 ng/mL for urine), a longer detection time (DT) was observed for plasma than for urine, especially after multiple doses, as plasma ISL corresponds to a slower terminal elimination phase. Increasing the current plasma ISL from 1 to 3 ng/mL—while keeping the current urine ISL at 100 ng/mL—could better align the plasma and urine DTs. As a limitation of this study, both Standardbred and Thoroughbred data were included, and further data collection is needed to fully ascertain potential breed‐specific effects. Moreover, this POP model also enabled relatively accurate Bayesian estimation of individual withdrawal times (WTs) from limited data. Clinicians could apply this Bayesian approach to making informed WT recommendations for horses when sufficient data is available. While existing non‐POP statistical models remain viable, they may require a more conservative approach to WT estimation than Bayesian methods.

## Graphical abstract

A meta‐analysis of flunixin concentrations in 65 horses from four countries estimated pharmacokinetic parameters. Considering the estimated plasma‐to‐urine ratio, raising the plasma International Screening Limit (ISL) from 1 to 3 ng/mL while keeping the urine ISL at 100 ng/mL could reduce the difference in detection times between the two matrices.

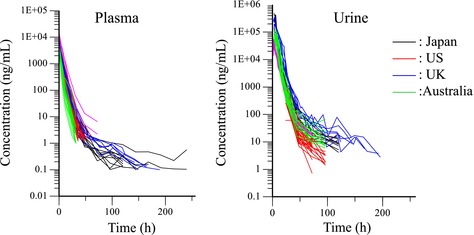

## 1 Introduction

Flunixin meglumine (FM) is one of the most commonly used nonsteroidal anti‐inflammatory drugs (NSAIDs) for managing inflammation and pain associated with soft tissue disorders in horses. It is also considered effective in controlling abdominal pain and is routinely used as part of standard care for equine colic and ocular pain related to ophthalmic diseases [1, 2, 3, 4].

Regulatory authorities for horse racing have adopted different frameworks for doping and medication control. For doping agents such as anabolic steroids, their objective is to detect even trace amounts of drugs using highly sensitive methods [5, 6, 7]. In contrast, for medications such as flunixin, irrelevant plasma concentration (IPC), and irrelevant urine concentration (IUC) are determined to define screening limits (SLs), thereby ensuring fair competition while providing proper veterinary care [5, 8]. These thresholds are typically determined using the Toutain modeling method, based on pharmacokinetic/pharmacodynamic (PK/PD) analysis, as recommended by the European Horserace Scientific Liaison Committee (EHSLC) [7]. Similar approaches are followed by other authorities, including the Horseracing Integrity and Safety Authority (HISA) in the United States, the Japan Racing Association, the Hong Kong Jockey Club, and Racing Australia [8, 9].

The time from drug administration until drug concentrations fall below the SL is called the detection time (DT). The EHSLC defines DT as the time interval between the last drug administration and the point at which the observed urine (or plasma) concentrations are below the SL for all horses enrolled in a trial [10]. DT determinations are usually based on single‐dose administrations in a sample size of 6–8 horses. It is important to understand that the DT reported by the EHSLC serves only as preliminary guidance to help prescribing veterinarians determine their own withdrawal time (WT), and that actual WT in clinical cases may significantly exceed the experimental DT. Various factors influence observed DT, including the number of horses in the study, dosing regimen, administration route, formulation, breed, age, sex, and especially the between‐subject variability (BSV) [10].

To assist veterinarians in determining individualized WTs based on a drug's DT, it may be beneficial to consider additional information—particularly regarding BSV and relevant covariates—using a population pharmacokinetic (POP PK) model. The goal of POP PK analysis is to estimate BSV across a large and diverse horse population and to identify key covariates such as breed, sex, and age that may influence WT [10]. In addition, Bayesian estimation has been proposed as a tool for veterinarians to more confidently calculate WT for individual horses—referred to as individual Bayesian withdrawal time (IBWT)—by incorporating blood or urine concentration data and horse‐specific covariates into an ad hoc generic POP model [11].

A POP PK analysis of flunixin in 20 horses found that the computed DT was longer in plasma than in urine, under the International Federation of Horseracing Authorities (IFHA) international screening limits (ISLs) [12]. This discrepancy likely arises from a mismatch between the observed experimental steady‐state urine/plasma ratio (Rss) of 36.5 and the assumed operational Rss of 100, derived from the urine and plasma SLs of 100 and 1 ng/mL, respectively [13, 14]. Additionally, plasma DT after multiple administrations of 1.1 mg/kg q 24 h was longer than the 144‐h DT limit set by IFHA [12, 15]. Where feasible, it would be desirable to align the plasma and urine DTs more closely so that the results from the two matrices are comparable. Since the Rss data was based on 20 thoroughbred horses from a single country, it was theorized that broader datasets could enhance the international relevance of these findings for making harmonized decisions. A population meta‐analysis using pharmacokinetic data from larger, multicountry samples could help assist this need, provided that sufficiently large datasets are available.

Therefore, this population analysis aimed to develop a flunixin disposition model using data from four countries to support international harmonization of SLs. In addition, this meta‐analysis aimed to demonstrate how such a POP model can assist veterinarians in establishing individualized WTs for a given horse using Bayesian estimation.

## 2 Materials and Methods

### 2.1 Data Collection

Demographic and pharmacokinetic data collected from 65 horses across four countries—Japan, the USA, the UK, and Australia—was considered to prepare the framework of this meta‐analysis. Some of these data—particularly those from Japan, the USA, and Australia—have already been published [12, 16, 17, 18]. This meta‐analysis was focused only on the unchanged form of flunixin; the hydroxylated metabolite (–OH) was not included. Intravenous (IV) doses of FM in horses ranged from 0.84 to 1.1 mg/kg. Of the total of 65 horses, 10 Japanese horses received multiple doses of FM for 5 days; the remaining 55 horses received a single FM IV administration (Table 1).

**TABLE 1:** Drug administration protocols, LOQ of analytical techniques, and demographic characteristics of the horses (dataset for the present meta‐analysis).

Countries		No. horses (*n*)		Dose of flunixin (mg/kg or mg/horse)
		Sex		
	Trade name	Age	LOQ (ng/mL)	
	Route of administration	Breed	Plasma	
	(company, place)	Body weight	U = urine	
Japan	Banamine® 5% injection	*N* = 10	P: 0.1	1.1 mg/kg
	IV	Female	U: 3.0	single
	(DS Pharma Animal Health Co. Ltd, Osaka, Japan)	4–10 years		
		Thoroughbred		
		428–506 kg		
Japan	Banamine® 5% injection	*N* = 10	P: 0.1	1.1 mg/kg
	IV	Female	U: 3.0	q 24 h 5 days
	(DS Pharma Animal Health Co. Ltd, Osaka, Japan)	4–9 years		
		Thoroughbred		
		442–530 kg		
USA	Banamine®	*N* = 16	P: 1	1.1 mg/kg
	IV	8 females, 8 gelding	U: 0.5	single
	(Merck Animal Health, Whitehouse Station, NJ, USA)	5–8 years		
		Thoroughbred		
		491–626 kg		
USA	Banamine®	11	P: 1.0	500 mg/horse
	IV	3 females, 8 gelding	U: 0.5	single
	(Merck Animal Health, Whitehouse Station, NJ, USA)	3–8 years		
		Thoroughbred		
		469–592 kg		
UK	Flunixin Injection	6	P: 1.0	1.1 mg/kg
	IV	4 males, 2 gelding	U: 0.5	single
	(Norbrook Laboratories Ltd. Newry, Northern Ireland)	10–22 years		
		Thoroughbred		
		434–537 kg		
Australia	Flunix ®	12	P: 1.0 U: 1.0	1.1 mg/kg
	IV	Gelding		single
	(Bomac Animal Health Pty Ltd., Hornsby NSW, Australia) (now defunct)	4–12 years		
		Standardbred		
		421–524 kg		

In Japan, the USA, and Australia, analytical methods for detecting flunixin in plasma and urine have been previously reported [12, 16, 17, 18]. In the UK, flunixin concentrations in plasma and urine are measured using an LC–MS/MS system (Waters Quattro Premier). Calibration curves were prepared from samples at nine concentrations (0, 0.1, 0.5, 1, 5, 10, 25, 50, and 100 ng/mL) in plasma and seven concentrations (0, 10, 25, 50, 100, 250, 500, and 1000 ng/mL) in urine. Accuracy and precision were assessed using flunixin‐spiked plasma and urine quality control samples, analyzed using six replicates at three concentrations (0.75, 7.5, and 75 ng/mL in plasma and 15, 75, and 750 ng/mL in urine). For all control levels in both matrices, the coefficient of variation (%CV) (for precision) and relative error (for accuracy) were within 10% of the nominal concentrations.

### 2.2 Data Analysis

Data analysis was carried out using Phoenix® WinNonlin® 8.5 (Certara, Princeton, New Jersey, USA). The first step was to perform a noncompartmental analysis (NCA) to obtain an initial estimate of basic pharmacokinetic parameters such as plasma clearance, volume of distribution, and area under the curve (AUC) using the model (200–202 linear trapezoidal rule). While NCA is sometimes considered a simple approach, it remains a standard first step in population modeling to provide reliable starting values for more complex analyses, ensuring model convergence with large datasets. These parameters were used as the starting values for the comprehensive POP PK model, which was developed using a nonlinear mixed‐effects model (NLMEM).

**FIGURE 1:**
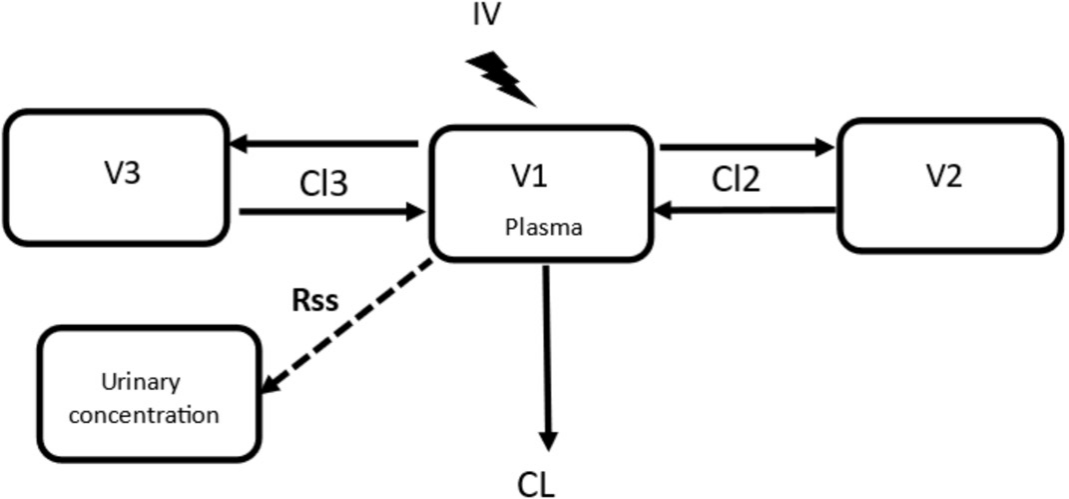
Diagram of the three‐compartment model. Rss: pseudo‐equilibrium urine‐to‐plasma ratio.

The limits of quantification (LOQ) for the analytical techniques are listed in Table 1. Flunixin concentrations below the LOQ that were encountered in less than 5% of the corresponding data were excluded from this analysis [19, 20]. Two models—a two‐compartment and a three‐compartment model—were compared using the likelihood ratio test, and the three‐compartment model was selected (Figure 1). Parameterization was in terms of plasma clearance (Cl), intercompartmental clearance(s) (Cl2 and Cl3), and volume(s) of distribution (V), with V1, V2, V3, Cl, Cl2, and Cl3 being the primary estimated parameters. Flunixin concentrations in the plasma and urine were analyzed simultaneously. Plasma flunixin concentration is known to be the driving factor that determines urine flunixin concentration, ensuring a priori parallelism between flunixin decay in plasma and urine [12]. Accordingly, the pseudo‐equilibrium urine‐to‐plasma ratio, Rss (U/P), of the flunixin concentration was estimated by adding an equation expressing the urine concentration as proportional to that in the plasma. From the plot of plasma and urine concentrations, only urine data collected 24 h after administration was considered to ensure pseudo‐distribution equilibrium, supporting plasma and urine concentration parallelism.

In the NLMEM, the random component (random effect) describes the variability around the fixed parameters of the structural model. BSV was modeled using an exponential model of the form (for plasma clearance):

(1) $$\theta_{\text{clearance}\_i}=\theta_{\mathrm{tv}\_\text{clearance}}\times \mathrm{Exp}\left(\eta_{i}\right)$$

where *θi* (theta) is the value of plasma clearance in the *i* ^th^ horse, $\theta_{\mathrm{tv}\_\text{clearance}}$ is the typical population value of theta (V1, V2, V3, Cl, Cl2, Cl3, Rss), and $\eta_{i}$ is the deviation associated with the *i* ^th^ horse from the corresponding theta population value. Thus, the variability among horses was estimated from their individual η values. The distribution of η was assumed normal with a mean of 0 and a variance ω [2].

To report interindividual variability as a coefficient of variation, Equation 2 was used to convert variance terms (ω [2]) into the coefficient of variation (CV%).

(2) $$\mathrm{CV}\left(\%\right)=100\times \sqrt{\exp\left(\omega^{2}\right)-1}$$

Shrinkage of the random effects ( η) towards the mean was described as:

(3) $$\text{shrinkage}=1-\frac{\mathrm{SD}\left({\mathrm{EBE}}_{\eta }\right)}{\omega }$$

When the shrinkage of η was >0.3, the data did not allow for a robust estimation of this random component. Because there were no parameters associated with shrinkage for η >0.3, a random component was added to all parameters. For the present analysis, a full OMEGA matrix was considered, meaning that both variance and covariance terms were estimated. The residual error model was an additive plus a multiplicative (proportional) model of the form.

(4) $$C\left(t\right)=f\left(\theta ,\text{Time}\right)\times \left(1+\varepsilon_{1}\right)+\varepsilon_{2}$$

with ε_1_ being the multiplicative error term having a mean of 0 and a variance noted *σ*1:

$$\varepsilon 1\approx N\left(0,\sigma 1^{2}\right)$$

and ε_2_ being the additive error term having a mean of 0 and a variance noted *σ*2:

$$\varepsilon 2\approx N\left(0,\sigma 2^{2}\right)$$

A quasi‐random parametric expectation‐maximization (QRPEM) engine was used to estimate the parameters. The parameter precision was estimated using a bootstrap tool (*n* = 50). The influence of covariates (country, age, body weight [BW], breed, and sex) was also assessed. The stepwise covariate search mode of the Phoenix NLMEM was used to define statistically significant covariates for each structural parameter. The stepwise forward or backward addition or deletion of covariate effects (by adding them one at a time) determined the improvement in the final model based on the Bayesian information criterion (BIC). The BIC is defined as:

(5) $$\mathrm{BIC}=-2\cdot \text{In}\left(L\right)+k\;\text{In}\left(n\right)$$

where *L* is the maximum likelihood of the model, *k* is the number of parameters in the model, and *n* is the number of data points [21]. BIC value of 6.635 and 10.823 were used to add and delete covariates respectively, these two values being the χ^2^ critical values for *p* = 0.01 and 0.001 for a degree of freedom of 1. As there was no model with a BIC <10.0 compared with models without covariates, no covariates were included in the final model [21].

### 2.3 Estimation of EPC, IPC, and IUC

The effective plasma concentration (EPC), IPC, and IUC were calculated using the Toutain approach [7]. Plasma clearance was used to calculate the EPC using the following equation:

(6) $$\mathrm{EPC}=\frac{\text{Dose}\;\mathrm{per}\;24\;h}{\text{Clearance}\;\mathrm{per}\;24\;h}$$

A dose of 1.1 mg/kg q 24 h and the typical clearance estimated by the model were used to compute the EPC. The IPC was obtained after dividing the EPC by the selected uncertainty factor (500), and the IUC was obtained after multiplying the flunixin IPC by the corresponding typical value (theta) of Rss estimated using the model.

(7) IPC=EPC/500

(8) IUC=IPC×tvRss

The resulting DTs were compared with the current SLs and with those calculated from the IPCs and IUCs derived from the POP model.

### 2.4 Estimation of DTs

We first estimated the DTs using IFHA ISL in plasma (1 ng/mL) and urine (100 ng/mL) [13, 14]. We then estimated the DT for the new candidate SLs calculated from the IPC and IUC, as estimated in this study, following the EHSLC definition. In addition, the DTs of SLs indicated by HISA (4 ng/mL in plasma) [22] and the Racing Medication and Testing Consortium (RMTC) (5 ng/mL in plasma) [23] were also estimated. As a second approach to estimate DTs, we used the Phoenix NCA engine to calculate the individual DT for the IFHA ISLs, candidate SLs, HISA SL, and RMTC SL for each horse. As a third approach, a Monte Carlo simulation was used to generate the plasma and urine concentrations of a virtual population of 5000 horses using individual predictions (IPRED) ( η as estimated), corresponding to 1.1 mg/kg single and multiple (q 12 h and q 24 h for 5 days) administrations. Using this meta‐population, the distribution of 5000 individual DTs corresponding to IFHA ISLs, candidate SLs, HISA SL, and RMTC SL was calculated. The difference between the plasma and urine DT of each individual was also calculated for the 5000 horses and the degree of agreement was assessed using Bland and Altman plots [24]. These data were then analyzed using the Phoenix statistical tool to compute the quantiles of interest (5^th^, 10^th^, 25^th^, 50^th^, 75^th^, 90^th^, and 95^th^). Finally, we explored the SL for plasma and urine to minimize the differences between the DTs calculated.

### 2.5 Bayesian Estimates of DTs for a New Horse

The plasma and urine concentrations selected for this Bayesian prediction were those observed in 55 horses (single dose) and 10 Japanese horses that received multiple doses. Two scenarios were used to simulate a situation in which a veterinarian collects blood and urine samples from a horse for Bayesian estimate.

1‐“Rich” Scenario: In this scenario, three blood samples were collected at 9, 24, and 48 hours after IV administration of FM at a dose of 1.1 mg/kg, along with a urine sample collected at 48 hours.

2‐“Sparse” Scenario: In this scenario, a single blood sample was collected 24 hours after either a single IV FM administration or after the fifth dose in the multiple‐dosing regimen.

By inputting the concentrations of these two scenarios into the population model, Bayesian estimates of plasma and urine concentration curves were obtained using the post hoc option of the model. In this framework, the population pharmacokinetic model served as the prior distribution for individual parameters, while the observed concentrations from each horse (sparse or rich sampling scenarios) were used to update these priors to obtain posterior distributions, according to Bayes' theorem. This approach enabled individualized WT predictions that incorporated both prior knowledge from the population model and individual‐specific data, thereby increasing the certainty compared to non‐Bayesian methods [11]. DTs for the current IFHA ISL in plasma (1 ng/mL) and urine (100 ng/mL) were estimated based on Bayesian prediction and compared to the known individual DTs estimated by the NCA engine using all plasma/urine concentrations for each horse.

## 3 Results

**FIGURE 2:**
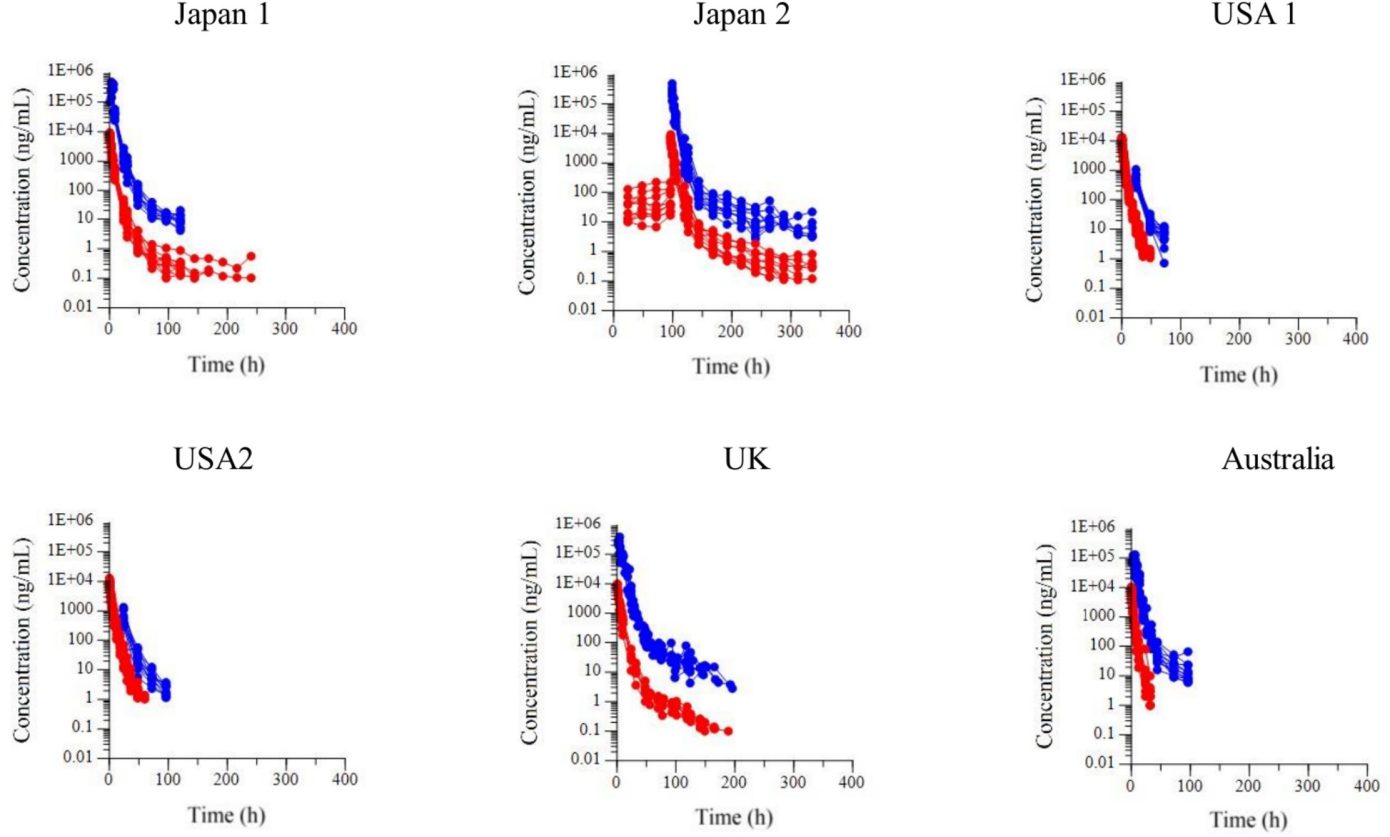
Semilogarithmic spaghetti plots of the disposition curves of plasma (red) and urine (blue) flunixin concentration after administration of FM for each experiment in four countries. All experiments except for Japan 2 were single IV dose experiments, and Japan 2 was a multiple IV dose experiment for 5 days at 24‐h intervals.

**FIGURE 3:**
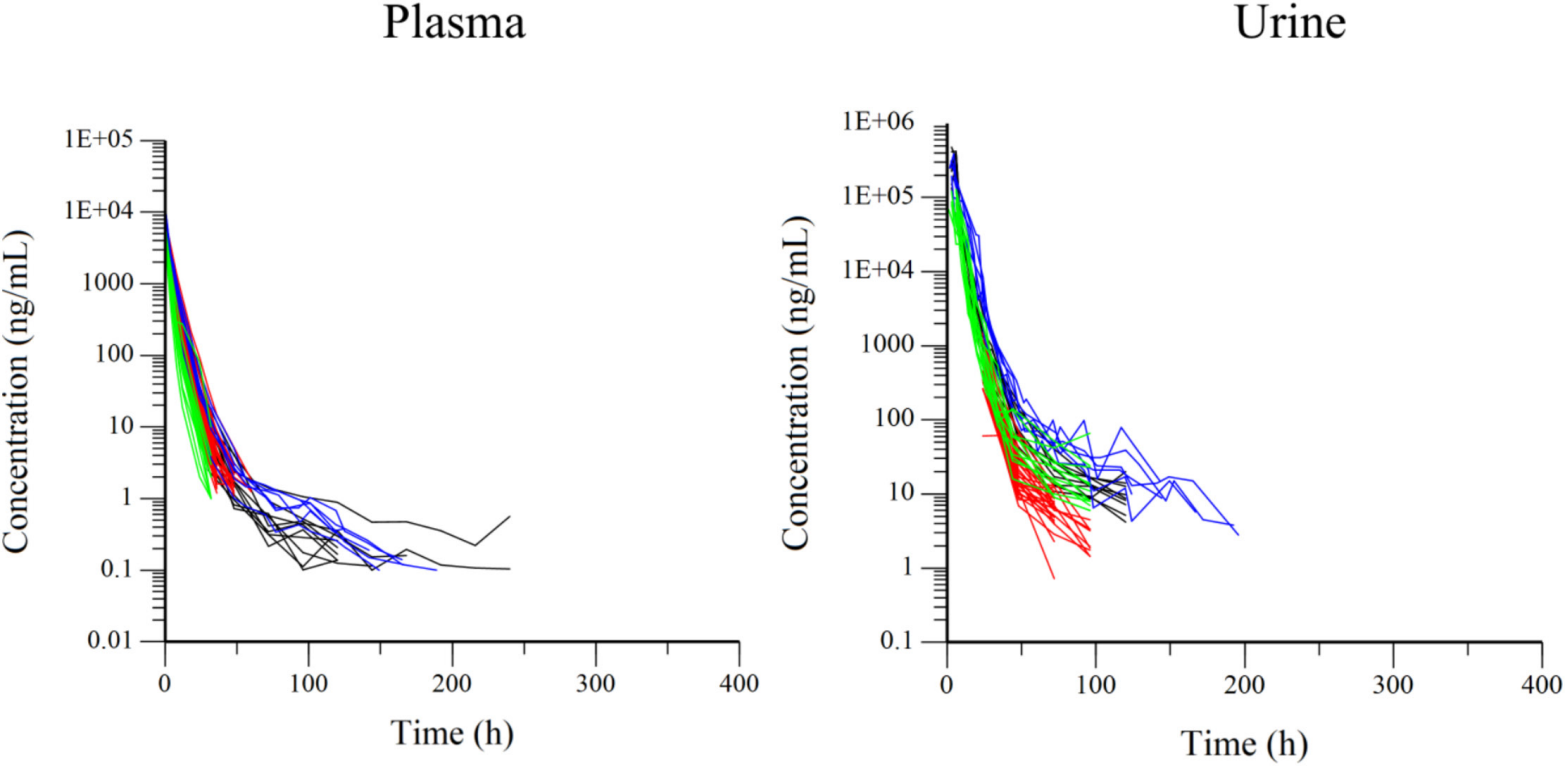
Semilogarithmic spaghetti plots of the disposition curves of plasma (left) and urine (right) flunixin concentration after single administration (black = Japan; red = US; blue = UK; green = Australia) by IV route for a scaled dose of 1.1 mg/kg.

Figure 2 shows the semilogarithmic plots of the disposition curves for the plasma and urine flunixin concentrations for each experiment. Figure 3 compares the disposition curves of plasma and urine flunixin after a single‐dose administration and shows the influence or lack thereof of the LOQ on the detection of the very late terminal phase. The results of the parameters estimated by NCA analysis of the plasma of the 65 horses are shown in Supplemental File S1.

Modeling with the NLMEM showed that body weight and age were likely covariates to be considered in explaining the intersubject variability in clearances, volume of distribution, and Rss (Supplemental Files S2 and S3). However, due to imbalances in the data structure between countries, these covariates were not retained in the final model because their effects were confounded by those of the country of origin of the data (see Discussion section).

**FIGURE 4:**
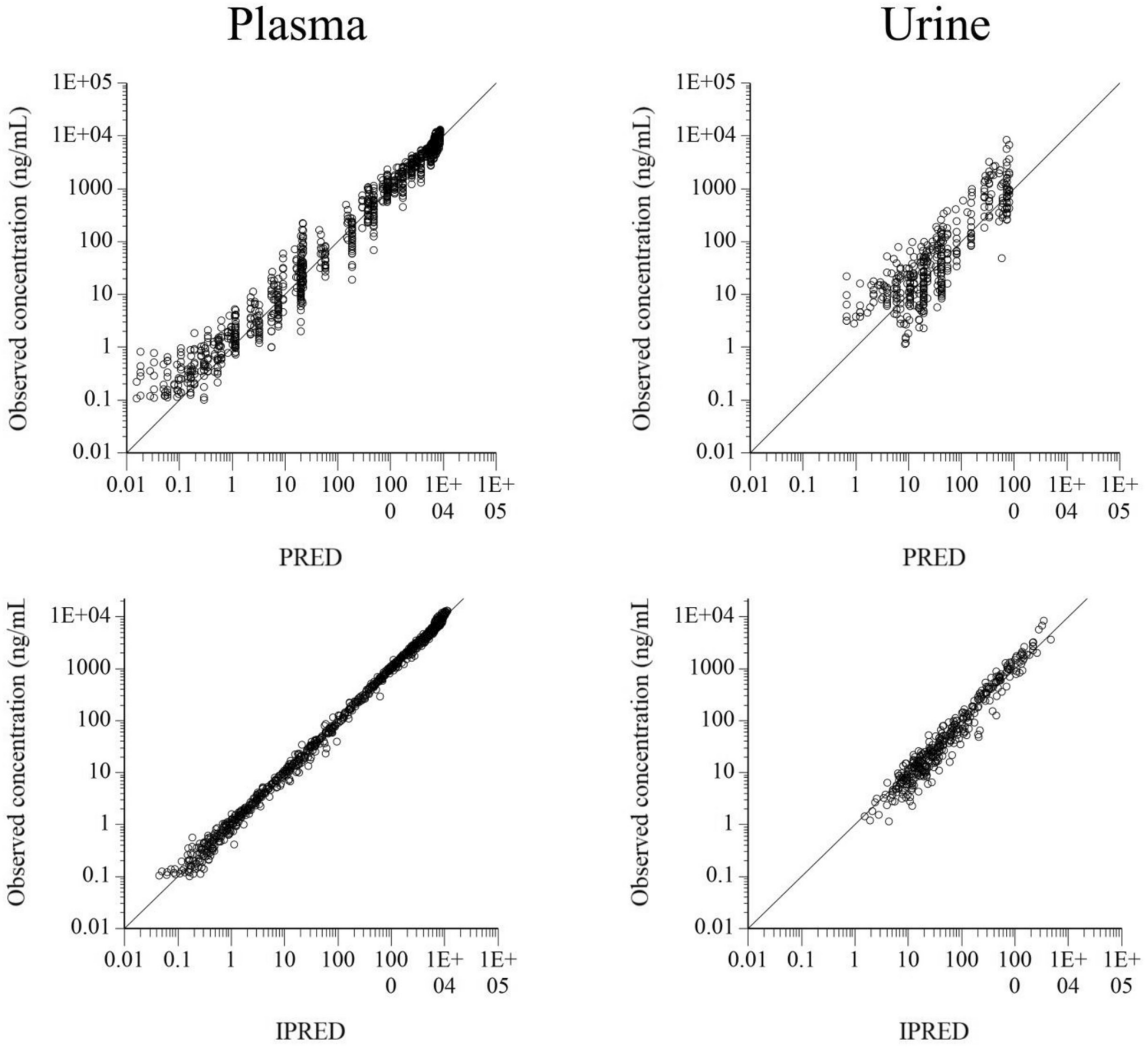
Logarithmic plots of observed flunixin concentrations in plasma (left) and urine (right) versus population (PRED) (top plots) and individual predictions (IPRED) (bottom plots).

**FIGURE 5:**
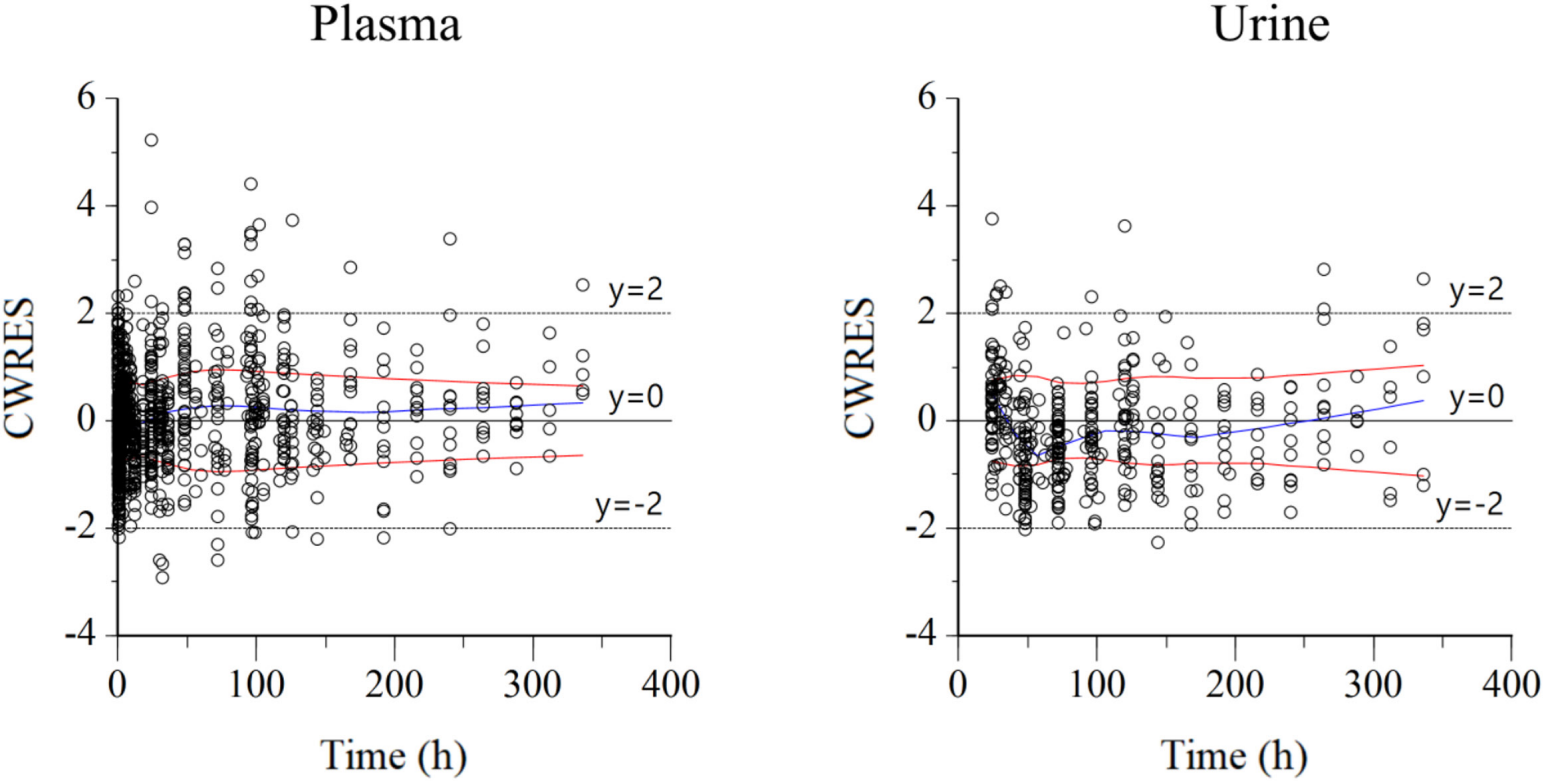
Conditional weighted residuals (CWRES) versus time plot for plasma (left) and urine (right). Values of CWRES should be approximately *N* (0, 1) and hence concentrated between *y* = −2 and *y* = +2. Inspection of the figure indicates that data were evenly distributed about zero and that the trends (as given by the blue line and the red line with its negative reflection) did not show any fanning, thus indicating no bias in the structural model.

**FIGURE 6:**
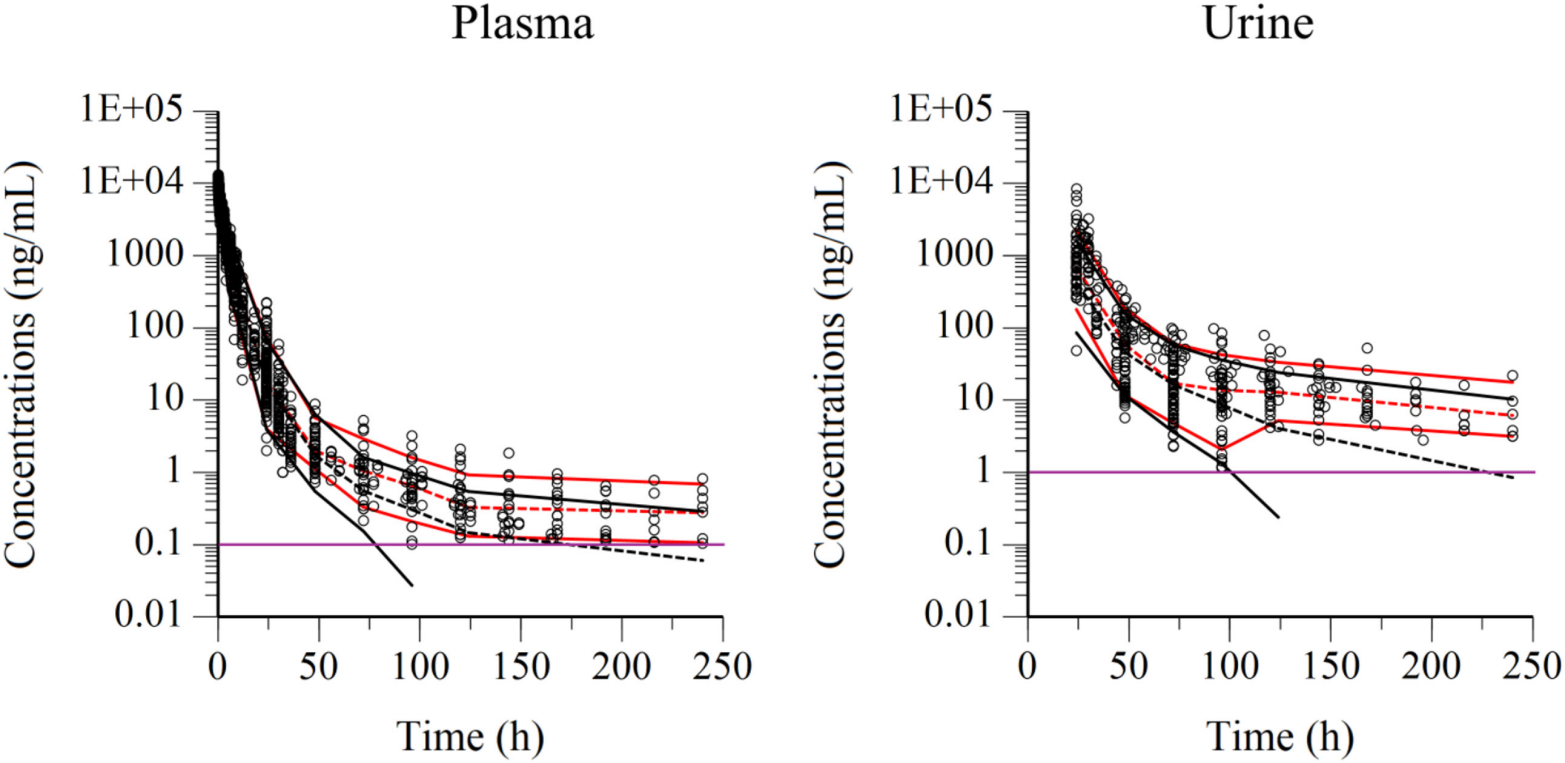
Visual predictive check of observations versus time after dose in plasma (left) and urine (right) in the case of a single dose of 1.1 mg/kg BW. The observed and predicted 10^th^ and 90^th^ percentiles are indicated via solid red and black lines respectively. The observed and predicted 50^th^ percentiles (median) are indicated via red and black broken lines respectively. Blue dots represent individual raw data. Purple lines were LOQ of the model.

For the final POP model, logarithmic plots of the observed drug plasma and urine concentrations versus population prediction (PRED) and IPRED are shown in Figure 4. The data were evenly distributed around the line of identity, indicating no major bias in the population components of the model. The plot of the conditional weighted residuals versus time indicated that the residuals were randomly scattered around zero with no systematic trend, supporting the residual error model selection (Figure 5). A Visual Predictive Check ensured that the simulated data was consistent with the observed data (Figure 6). Plots of the dependent variables in the plasma and urine and individual predicted curves after dose administration in 65 horses are shown in Supplemental File S4.

Bootstrap estimates of the typical values of the primary structural parameters of the model (theta), secondary parameters, and their associated coefficients of variation as a measure of the precision of their estimation are given in Table 2. The typical value of Rss was 35.9. The interindividual variability computed using Equation 2 in Table 3 and BSV for Rss was high, with a coefficient of variation of 99.0%. An EPC of 967.9 ng/mL (95% CI: 895.0–1021.5 ng/mL) was computed after 1.1 mg/kg BW q 24 hours. The flunixin IPC was estimated at 1.9 ng/mL (95% CI: 1.8–2.0 ng/mL), and the IUC was estimated at 70.2 ng/mL (95% CI: 57.4–83.6 ng/mL) (Table 4).

**TABLE 2:** Bootstrap estimates of typical values of the primary structural parameters and secondary parameters of flunixin after IV administration in horses, as obtained with a three‐compartment model.

	Estimate	Units	Std_err	CV%	2.5% CI	97.5% CI
**Primary parameters**						
tvV1	121.00	mL/kg	3.39	2.79	117.06	129.02
tvV2	8.90	mL/kg	0.71	7.97	7.83	10.50
tvV3	31.68	mL/kg	1.97	6.24	28.56	35.48
tvCl	47.36	mL/(kg*h)	1.90	4.00	44.87	51.21
tvCl2	0.22	mL/(kg*h)	0.03	13.15	0.19	0.29
tvCl3	7.54	mL/(kg*h)	0.91	12.00	6.15	9.51
tvRss	35.93	scalar	4.23	11.62	28.66	45.29
tvCMultStdev	0.15		0.04	28.61	0.12	0.17
tvCMultStdev_urine	0.42		0.03	6.33	0.38	0.48
stdev0	0.12	ng/mL	0.02	15.81	0.09	0.15
stdev1	0.11	ng/mL	0.32	147.88	‐0.06	0.99
**Secondary parameters**						
Half‐life alpha	1.4	h	0.1	5.5	1.2	1.5
Half‐life beta	3.8	h	0.2	4.6	3.3	4.0
Half‐life gamma	28.4	h	3.3	11.5	22.1	34.3
Vss	161.6	L/kg	4.2	2.6	155.8	171.0
MRT (IV)	3.4	h	0.1	3.2	3.2	3.6

Abbreviations: Cl: plasma clearance; Cl2: distribution clearance from central compartment to compartment 2; Cl3: distribution clearance from central compartment to compartment 3; CMultStdev and CMultStdev_urine: multiplicative component of the error for plasma and urine, respectively, that should be read as a CV = 15% and 42%; Rss: urine‐to‐plasma ratio in pseudo‐equilibrium condition; MRT: mean residence time; stdev0 and stdev1: additive component of the residual error model for plasma and urine, respectively; V1: volume of the central compartment; V2: volume of the superficial peripheral compartment; V3: volume of the deep peripheral compartment; Vss: distribution volume at steady‐state.

**TABLE 3:** Estimates of the random effects (full variance/covariance matrix) and shrinkage.

Label	nV1	nCl	nCl2	nCl3	nV2	nV3	nRss
Omega							
nV1	**0.032**						
nCl	0.030	**0.085**					
nCl2	–0.016	0.050	**0.303**				
nCl3	–0.027	0.030	0.237	**0.352**			
nV2	0.047	0.051	0.181	0.049	**0.335**		
nV3	0.011	0.032	0.117	0.190	0.070	**0.140**	
nRss	0.083	0.151	0.030	–0.035	0.098	0.000	**0.683**
Correlation							
nV	1.000						
nCl	0.574	1.000					
nCl2	–0.166	0.313	1.000				
nCl3	–0.256	0.171	0.726	1.000			
nV2	0.457	0.302	0.567	0.143	1.000		
nV3	0.166	0.298	0.567	0.857	0.324	1.000	
nRss	0.562	0.626	0.066	–0.072	0.204	0.000	1.000
Shrinkage	0.07	0.01	0.17	0.21	0.12	0.18	0.04
BSV (%)	18.1	29.8	59.5	64.9	63.1	38.7	99.0

*Notes:* Between‐subject variability (BSV %) was computed using Equation 2.

Variance terms are mentioned in bold. Out diagonal figures of OMEGA represent covariance terms. Shrinkage was rather low, indicating that data were rich enough allowing a proper estimate of the BSV that was relatively large due to the heterogeneity of the horses data set. The variability for Rss was the highest, with a coefficient of variation of 99%.

**TABLE 4:** Estimation of effective plasma concentration, irrelevant plasma concentration, and irrelevant urine concentration using the Toutain model approach.

Parameter (units)	Estimate	Std_err	CV%	2.5% CI	97.5% CI
EPC (ng/mL)	967.9	38.07	3.9	895.0	1021.5
IPC (ng/mL)	1.9	0.08	3.9	1.8	2.0
IUC (ng/mL)	70.2	6.67	9.5	57.4	83.6

**FIGURE 7:**
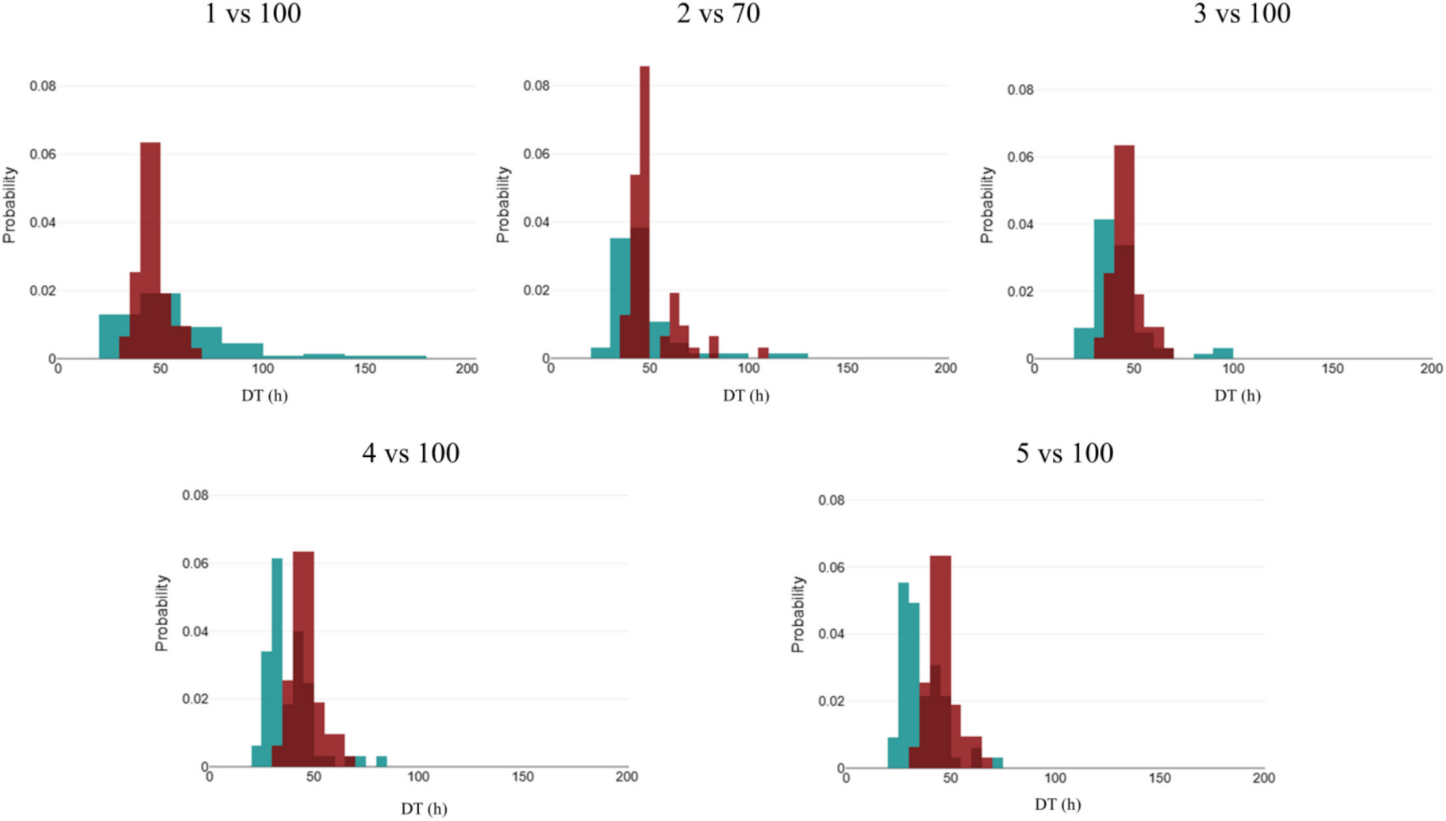
Distribution of plasma DT (green) versus urinary DT (brown) for 5 sets of SLs in plasma (1 to 5 ng/mL) and 70 or 100 ng/mL for urine in 65 horses.

Several SLs estimated based on the IPC and IUC were compared with IFHA ISL (1 ng/mL in plasma and 100 ng/mL in urine), HISA SL (4 ng/mL in plasma), and RMTC SL (5 ng/mL in plasma). The first candidate SLs were those obtained with the IPC (2 ng/mL rounded from 1.9 ng/mL) and IUC (70 ng/mL rounded from 68.4 ng/mL) estimated in the present study. We also explored an SL of 3 ng/mL in plasma, calculated by dividing the current ISL in urine (100 ng/mL) by the Rss (35.9) obtained in this study. DTs for the current IFHA ISL in plasma were the longest at 168 h in the Japanese multiple administration experiment, and the plasma DT was longer than the urine DT for ISL in Japan, the US, and Australia (Table 5). In individual DTs estimated by interpolation using the NCA engine, the longest observed DT for IFHA ISL was 104 h for plasma and 64 h for urine after a single administration, and no horse exceeded the recommended DT of 144 hours published on the IFHA website [15]. After multiple administrations, the longest DT observed for IFHA ISL was 167 h for plasma and 69 h for urine, with one horse exceeding the IFHA SL in plasma (Table 6). The distribution of plasma DT versus urinary DT estimated by the NCA engine for four SLs in plasma (1, 3, 4, and 5 ng/mL) versus the current ISL in urine (100 ng/mL) and IPC (2 ng/mL) versus IUC (70 ng/mL) is depicted in Figure 7. The largest difference between plasma and urine was 123.5 h (ID: 20 for multiple administrations) for ISL (1 vs. 100 ng/mL), and the frequency of longer DTs in plasma compared to urine decreased as the plasma SL increased. For plasma and urine SLs of 2 versus 70 ng/mL or 3 versus 100 ng/mL, corresponding to Rss close to the computed Rss of 35.9, the model values of the urinary and plasma DT distributions overlap, minimizing the difference between plasma and urine DT. Bland–Altman plots to assess agreement between the two DTs in plasma vs. urine explored in five SLs and estimates of individual differences in plasma and urine DTs are given in Supplemental File S5 and S6.

**TABLE 5:** Detection times (h) obtained with the EHSLC definition for different groups of horses analyzed in this meta‐analysis.

		Dose	Plasma SL (ng/mL)					Urine SL (ng/mL)	
Countries	No. horses	mg/kg or mg/horse	1	2	3	4	5	70	100
Japan	10	1.1 mg/kg single	120	72	72	72	48	72	72
Japan	10	1.1 mg/kg q 24 h* 5 days	168	144	72	72	72	120	72
USA	16	1.1 mg/kg single	72	72	48	48	48	48	48
USA	11	500 mg/horse single	72	60	60	48	48	48	48
UK	6	1.1 mg/kg single	94	56	56	56	56	75.92	73.15
Australia	12	1.1 mg/kg single	144	72	72	48	48	72	72

Note: Different sets of screening limits (SL) for plasma and urine were considered, i.e., current international IFAH ISLs and candidate SLs, to obtain better alignment between the DTs in plasma and urine.

**TABLE 6:** Estimation of individual DTs in plasma and urine computed by interpolation by the Phoenix NCA engine for the current plasma and urine ISLs endorsed by IFHA (1 ng/mL for plasma and 100 ng/mL for urine) and for others candidate SLs. DT in urine were not calculated (NC) by the Phoenix NCA engine in 2 horses due to lack of data.

			Plasma SL (ng/mL)					Urine SL (ng/mL)	
ID	Nation	Dosing	1	2	3	4	5	70	100
1	Japan	1.1 mg/kg single	51.7	46.8	45.5	44.2	42.9	47.8	47.3
2	Japan	1.1 mg/kg single	47.4	45.1	42.9	40.6	38.4	46.3	45
3	Japan	1.1 mg/kg single	68.2	61.8	55.4	49	45.7	64.6	57.2
4	Japan	1.1 mg/kg single	58.3	45.9	40.9	35.8	30.8	49.1	45.8
5	Japan	1.1 mg/kg single	62.9	47.5	45.5	43.4	41.4	47.8	47
6	Japan	1.1 mg/kg single	60.5	47	42.9	38.8	34.6	47.7	47.1
7	Japan	1.1 mg/kg single	47.5	35.6	29.6	28.8	28	44.6	40.7
8	Japan	1.1 mg/kg single	46.7	39.8	32.9	29.3	28.2	59.4	50.6
9	Japan	1.1 mg/kg single	71.4	63.6	55.8	48	46.9	63.9	58.9
10	Japan	1.1 mg/kg single	104.2	57.1	46.3	43.5	40.8	NC	NC
11	Japan	1.1 mg/kg q 24 h 5 days	71.9	47.3	44.5	41.7	38.9	47.6	46.8
12	Japan	1.1 mg/kg q 24 h 5 days	67.1	47.3	40.7	34.1	29.7	68.1	60.9
13	Japan	1.1 mg/kg q 24 h 5 days	89.1	66.7	55.3	47.4	45.7	47.9	46.8
14	Japan	1.1 mg/kg q 24 h 5 days	141.5	114.5	99.2	72.9	64.4	106.8	68.8
15	Japan	1.1 mg/kg q 24 h 5 days	83.8	59.4	47.5	46.1	44.8	62.6	52.7
16	Japan	1.1 mg/kg q 24 h 5 days	132	90.9	80.3	47.4	46.5	46.2	44.1
17	Japan	1.1 mg/kg q 24 h 5 days	135.8	83.3	69.5	65.8	62.1	47.7	46.7
18	Japan	1.1 mg/kg q 24 h 5 days	74.1	47.3	44.3	41.4	38.4	83.1	47.7
19	Japan	1.1 mg/kg q 24 h 5 days	88	70.2	63.7	57.2	50.6	47.9	47.5
20	Japan	1.1 mg/kg q 24 h 5 days	166.9	129.6	90.5	82.3	74.1	45.4	43.3
21	US	1.1 mg/kg single	40.7	35.9	33.8	31.8	29.9	42.6	39.7
22	US	1.1 mg/kg single	41.7	44.6	35	32.4	29.9	45.1	43.5
23	US	1.1 mg/kg single	43.6	41.3	35.3	33.4	31.6	43.6	41.3
24	US	1.1 mg/kg single	40.1	34.7	31.3	29.4	28.4	45.8	44
25	US	1.1 mg/kg single	53.6	47.6	45.5	42.7	39.8	42.6	39.7
26	US	1.1 mg/kg single	38.5	34.5	31.6	29.5	28.5	44.5	42.8
27	US	1.1 mg/kg single	39.8	35.8	33.7	31.7	29.9	46.2	45.2
28	US	1.1 mg/kg single	37.9	34.6	32.5	30.3	29.3	43.8	41.6
29	US	1.1 mg/kg single	43.6	38.4	34.6	32.8	31	46	45
30	US	1.1 mg/kg single	46.1	45.3	40.7	36.1	35	46.1	45.1
31	US	1.1 mg/kg single	41.8	37.7	35.8	34.3	32.8	46.3	45.2
32	US	1.1 mg/kg single	38.8	35.3	32.6	30	29.2	44.5	42.8
33	US	1.1 mg/kg single	48.8	46.2	42.5	38.8	35.8	45.3	44
34	US	500 mg/horse single	40.5	36.1	34.2	32.4	30.5	43.7	41.6
35	US	500 mg/horse single	40.4	36.7	34.6	34.3	32.3	46.8	46.1
36	US	500 mg/horse single	37	32.8	29.6	28.2	26.8	42.2	39.4
37	US	500 mg/horse single	43.8	44.6	39	35.4	34	45.7	43.9
38	US	500 mg/horse single	60.3	49.3	44.3	40	35.9	45.9	44.5
39	US	500 mg/horse single	56.2	49.4	46.5	44	41.6	42.5	39.9
40	US	500 mg/horse single	63.9	57.2	52.5	47.9	46.3	47.2	46.5
41	US	500 mg/horse single	61.5	42.5	34.3	31.1	29.4	NC	NC
42	US	500 mg/horse single	54.2	48.2	46.3	43.3	40.3	47.4	46.1
43	US	500 mg/horse single	49	43	37.2	34.8	33.4	47	46.4
44	US	500 mg/horse single	45.1	45	40.4	35.9	34.9	44.5	42.6
45	US	500 mg/horse single	49.2	47.7	45.2	42.7	40.2	45.9	44.8
46	US	500 mg/horse single	39.2	35.8	33.2	30.7	29.5	43.7	41.4
47	US	500 mg/horse single	46.1	42	35.9	34.7	33.5	47	46.5
48	UK	1.1 mg/kg single	81.8	47.8	46.3	44.8	43.3	64	50.1
49	UK	1.1 mg/kg single	91.6	55.7	53.2	50.6	48	62.9	54.4
50	UK	1.1 mg/kg single	91.6	55.5	48.9	47.1	46	69.8	50.4
51	UK	1.1 mg/kg single	70.8	53.2	47.3	44.8	42.3	72	60.6
52	UK	1.1 mg/kg single	48.3	41.9	35.8	31.6	30.6	56.6	47
53	UK	1.1 mg/kg single	74.2	55.5	47.1	45.3	43.6	82.2	64
54	Australia	1.1 mg/kg single	38.2	34.2	32	31	30	40.3	37.6
55	Australia	1.1 mg/kg single	36.6	32	30	28	26	37.5	33.5
56	Australia	1.1 mg/kg single	35.5	32	30	28	26	66.4	55.4
57	Australia	1.1 mg/kg single	30	24	23.3	22.6	21.9	36.9	33.7
58	Australia	1.1 mg/kg single	43.7	40.1	38	32	32	60.9	53
59	Australia	1.1 mg/kg single	32	30	28	26	24	38.4	35.3
60	Australia	1.1 mg/kg single	35.6	32	30.4	28.8	27.2	43.3	41
61	Australia	1.1 mg/kg single	35.8	32	30.9	29.7	28.6	39.6	37
62	Australia	1.1 mg/kg single	38.4	34.4	32	31.2	30.4	43.5	42.9
63	Australia	1.1 mg/kg single	39.2	35.5	33.3	32	31.3	45.3	43
64	Australia	1.1 mg/kg single	32	28	24	23.6	23.2	42.2	37.2
65	Australia	1.1 mg/kg single	32	30.9	29.7	28.6	27.4	43.5	41.8
	Mean/single dose (h)		50.0	42.0	38.1	35.6	33.8	48.8	45.0
	Mean/multiple dose (h)		105.0	75.7	63.6	53.6	49.5	60.3	50.5
	Max/single dose (h)		104.2	80	74.5	72	72	82.2	64
	Max/multiple dose (h)		166.9	129.6	99.2	82.3	74.1	106.8	68.8

The estimation of DTs in a virtual population of 5000 horses generated by MCS after a single IV flunixin dose of 1.1 mg/kg and q 24 and 12 h for 5 days of multiple administrations is reported in Table 7. For a single administration, average DTs (50% quantile) were 52 and 39 h for the current IFHA ISL, and the corresponding 95% quantiles were 84 and 57 h for plasma and urine, which is shorter than the 144 h suggested as DT by IFHA [15]. For q 24‐h multiple administration, the average DTs were 76 and 45 h for plasma and urine respectively, and the corresponding 95% quantiles were 157 h for plasma and 88 h for urine. The statistical distribution (percentiles) of individual DT differences (plasma DT‐urine DT) in a population of 5000 virtual horses generated by MCS after single and multiple administrations is shown in Table 8. For SL of IFHA (1 vs. 100 ng/mL for plasma and urine respectively), the DT difference between plasma and urine (percentile 95% of the difference distributions) can be up to 41.4 h after single administration, 101.9 and 125.6 h after q 24 and 12 h for 5 days multiple administrations respectively.

**TABLE 7:** Estimation of the DTs (h) in a virtual population of 5000 horses generated by MCS after a single IV FM dose of 1.1 mg/kg (upper), q 24 h 5 days multiple doses (central) and q 12 h 5 days multiple doses (bottom). DTs were calculated for plasma SLs of 1, 2, 3, 4, 5 ng/mL and urine SLs for 70 and 100 ng/mL. The table presents the critical DT values for percentiles ranging from 5% to 95% across various SLs in plasma and urine.

For single administration	Plasma SL (ng/mL)					Urine SL (ng/mL)	
Percentiles	1	2	3	4	5	70	100
5%	35.3	29.5	26.7	25.0	23.7	30.2	27.7
10%	38.4	31.8	28.9	27.1	25.7	32.6	30.0
25%	44.1	36.3	32.9	30.7	29.1	36.7	33.6
50%	51.9	41.8	37.5	35.0	33.2	42.4	38.5
75%	61.9	48.4	43.3	40.2	38.2	49.5	44.5
90%	75.3	56.0	49.2	45.8	43.3	58.4	51.2
95%	84.4	61.3	53.4	49.3	46.7	66.1	57.0

For q 24 h 5 days multiple administration	Plasma SL (ng/mL)					Urine SL (ng/mL)	
Percentiles	1	2	3	4	5	70	100
5%	41.4	32.7	29.0	26.7	25.0	32.4	29.4
10%	46.1	35.3	31.2	28.7	26.9	35.6	32.0
25%	57.3	42.0	36.4	33.2	31.2	41.7	36.9
50%	76.0	52.1	43.7	39.4	36.6	51.7	44.6
75%	103.6	68.3	54.1	47.3	43.6	68.6	55.7
90%	133.7	90.6	69.6	58.3	52.1	93.0	73.3
95%	156.9	107.5	82.8	68.7	59.6	110.5	88.2

For q 12 h 5 days multiple administration	Plasma SL (ng/mL)					Urine SL (ng/mL)	
Percentiles	1	2	3	4	5	70	100
5%	51.5	37.9	32.3	29.4	27.3	37.2	32.6
10%	59.0	42.3	35.9	32.3	30.0	41.5	36.2
25%	75.5	52.8	43.1	38.1	35.1	51.6	43.4
50%	103.8	71.9	56.2	48.2	43.3	69.8	56.5
75%	139.9	100.0	78.8	65.7	56.7	97.4	79.1
90%	182.1	133.0	105.8	89.3	76.8	131.6	108.2
95%	192.0	155.8	127.9	107.9	93.3	154.4	128.6

**TABLE 8:** Statistical distribution (percentiles) of individual DT differences (plasma DT‐urine DT) in a population of 5000 virtual horses generated by MCS after a single IV FM dose of 1.1 mg/kg (upper), q 24 h 5 days multiple doses (central), and q 12 h 5 days multiple doses (bottom).

For single administration	Differences between individual DT in plasma and corresponding DT in urine for 5 sets of SL in plasma (1 to 5 ng/mL) vs. urine (70 or 100 ng/mL)				
Percentiles	1 vs. 100	2 vs. 70	3 vs. 100	4 vs. 100	5 vs. 100
5%	−5.2	−23.5	−17.7	−20.2	−22.1
10%	−0.5	−15.5	−12.4	−14.7	−16.5
25%	5.6	−6.9	−6.0	−8.4	−10.1
50%	12.5	−0.3	−0.7	−3.1	−4.8
75%	22.1	5.9	4.4	1.6	−0.3
90%	33.3	11.7	9.2	5.9	3.8
95%	41.4	16.1	12.4	8.6	6.4

For q 24 h 5 days multiple administration	Differences between individual DT in plasma and corresponding DT in urine for 5 sets of SL in plasma (1 to 5 ng/mL) vs. urine (70 or 100 ng/mL)				
Percentiles	1 vs. 100	2 vs. 70	3 vs. 100	4 vs. 100	5 vs. 100
5%	−11.3	−51.3	−40.9	−46.0	−49.9
10%	−1.6	−35.2	−27.2	−32.0	−35.7
25%	11.2	−13.7	−11.0	−15.3	−18.3
50%	28.4	0.5	−0.4	−4.4	−7.2
75%	54.2	14.2	9.2	3.8	0.6
90%	82.2	32.7	22.2	13.1	8.1
95%	101.9	46.8	32.4	20.2	13.7

For q 12 h 5 days multiple administration	Differences between individual DT in plasma and corresponding DT in urine for 5 sets of SL in plasma (1 to 5 ng/mL) vs. urine (70 or 100 ng/mL)				
Percentiles	1 vs. 100	2 vs. 70	3 vs. 100	4 vs. 100	5 vs. 100
5%	−16.6	−66.4	−61.5	−70.9	−77.2
10%	−2.3	−47.0	−43.3	−51.6	−57.8
25%	16.0	−20.9	−18.7	−26.2	−31.1
50%	39.9	1.9	0.2	−6.4	−10.7
75%	71.1	24.3	18.1	8.1	2.2
90%	103.9	50.0	40.8	25.8	16.8
95%	125.6	70.6	59.9	42.2	29.5

For the Bayesian estimation of individual WT for the rich predictive scenario, the average of absolute error between DTs by NCA (true value) and the estimated Bayesian DTs was 9.2 ± 11.0 h in plasma and 7.5 ± 3.4 h in urine (Table 9). The Bayesian estimate was close to the observed DTs in plasma, but the Bayesian estimate was shorter than the observed DTs in US horses (ID 21–47). In the sparse scenario where the plasma sample was a single point, the Bayesian estimate showed similar values to observed DTs and the average of absolute error between DTs was 9.9 ± 11.7 h (Table 9 and Supplemental File S7). The plasma DT values for the rich and sparse scenarios were also close to each other.

**TABLE 9:** Bayesian estimates of the estimated plasma and urinary DTs versus the true observed value estimated by NCA engine in 65 horses. For rich scenario, Bayesian estimates were calculated with three plasma concentrations measured at 9, 24, and 48 h after dosing, along with a single urine concentration at 48 hours following a single and multiple FM administration. For sparse scenario, Bayesian estimates were calculated with one plasma concentration measured at 24 hours after dosing following a single and multiple FM administration

			Plasma SL: 1 ng/mL			Urine SL: 100 ng/mL	
ID	Nation	Dosing	Observed	Bayesian sparse	Bayesian rich	Observed	Bayesian rich
1	Japan	1.1 mg/kg single	51.7	60.3	53.1	47.3	40.6
2	Japan	1.1 mg/kg single	47.4	46.6	44.8	45	35.1
3	Japan	1.1 mg/kg single	68.2	58.1	63.9	57.2	50.8
4	Japan	1.1 mg/kg single	58.3	46.4	61.0	45.8	39.4
5	Japan	1.1 mg/kg single	62.9	50.0	63.4	47	39.3
6	Japan	1.1 mg/kg single	60.5	48.6	65.4	47.1	37.2
7	Japan	1.1 mg/kg single	47.5	42.7	45.8	40.7	35.3
8	Japan	1.1 mg/kg single	46.7	42.2	43.2	50.6	45.3
9	Japan	1.1 mg/kg single	71.4	66.3	101.8	58.9	55.4
10	Japan	1.1 mg/kg single	104.2	53.4	72.7	NC	NC
11	Japan	1.1 mg/kg q 24 h 5 days	71.9	68.4	65.1	46.8	38.5
12	Japan	1.1 mg/kg q 24 h 5 days	67.1	71.2	75.4	60.9	60.0
13	Japan	1.1 mg/kg q 24 h 5 days	89.1	90.1	98.2	46.8	43.0
14	Japan	1.1 mg/kg q 24 h 5 days	141.5	150.4	113.8	68.8	59.4
15	Japan	1.1 mg/kg q 24 h 5 days	83.8	94.3	84.5	52.7	48.5
16	Japan	1.1 mg/kg q 24 h 5 days	132	83.4	96.3	44.1	39.9
17	Japan	1.1 mg/kg q 24 h 5 days	135.8	109.7	133.7	46.7	43.8
18	Japan	1.1 mg/kg q 24 h 5 days	74.1	74.1	70.7	47.7	46.6
19	Japan	1.1 mg/kg q 24 h 5 days	88	103.4	112.0	47.5	44.1
20	Japan	1.1 mg/kg q 24 h 5 days	166.9	113.7	99.3	43.3	37.9
21	US	1.1 mg/kg single	40.7	42.6	42.9	39.7	30.9
22	US	1.1 mg/kg single	41.7	44.4	62.1	43.5	30.1
23	US	1.1 mg/kg single	43.6	46.2	50.6	41.3	31.3
24	US	1.1 mg/kg single	40.1	42.2	59.0	44	32.2
25	US	1.1 mg/kg single	53.6	55.6	64.4	39.7	34.0
26	US	1.1 mg/kg single	38.5	41.3	43.1	42.8	28.3
27	US	1.1 mg/kg single	39.8	45.0	46.6	45.2	31.9
28	US	1.1 mg/kg single	37.9	43.1	43.8	41.6	30.0
29	US	1.1 mg/kg single	43.6	57.7	50.7	45	34.6
30	US	1.1 mg/kg single	46.1	53.8	53.8	45.1	33.4
31	US	1.1 mg/kg single	41.8	50.8	52.7	45.2	33.7
32	US	1.1 mg/kg single	38.8	42.9	44.7	42.8	29.9
33	US	1.1 mg/kg single	48.8	56.0	55.2	44	33.3
34	US	500 mg/horse single	40.5	44.8	45.7	41.6	29.6
35	US	500 mg/horse single	40.4	48.6	50.5	46.1	31.9
36	US	500 mg/horse single	37	38.8	39.2	39.4	27.7
37	US	500 mg/horse single	43.8	50.3	53.7	43.9	34.7
38	US	500 mg/horse single	60.3	55.2	65.6	44.5	34.9
39	US	500 mg/horse single	56.2	60.0	63.5	39.9	33.1
40	US	500 mg/horse single	63.9	71.5	75.9	46.5	38.9
41	US	500 mg/horse single	61.5	42.6	60.7	NC	NC
42	US	500 mg/horse single	54.2	56.1	69.3	46.1	39.2
43	US	500 mg/horse single	49	51.0	51.2	46.4	34.2
44	US	500 mg/horse single	45.1	53.1	54.5	42.6	32.1
45	US	500 mg/horse single	49.2	62.1	59.6	44.8	33.0
46	US	500 mg/horse single	39.2	44.2	43.5	41.4	31.8
47	US	500 mg/horse single	46.1	50.1	48.8	46.5	33.5
48	UK	1.1 mg/kg single	81.8	55.2	66.1	50.1	46.8
49	UK	1.1 mg/kg single	91.6	69.1	67.9	54.4	51.8
50	UK	1.1 mg/kg single	91.6	56.4	84.6	50.4	52.3
51	UK	1.1 mg/kg single	70.8	60.1	78.9	60.6	51.7
52	UK	1.1 mg/kg single	48.3	44.8	46.5	47	43.3
53	UK	1.1 mg/kg single	74.2	61.0	71.6	64	65.7
54	Australia	1.1 mg/kg single	38.2	43.1	41.7	37.6	33.0
55	Australia	1.1 mg/kg single	36.6	37.9	36.8	33.5	33.6
56	Australia	1.1 mg/kg single	35.5	39.2	38.7	55.4	45.7
57	Australia	1.1 mg/kg single	30	29.1	29.7	33.7	29.7
58	Australia	1.1 mg/kg single	43.7	76.8	68.9	53	45.4
59	Australia	1.1 mg/kg single	32	35.6	33.8	35.3	28.1
60	Australia	1.1 mg/kg single	35.6	39.6	38.6	41	35.9
61	Australia	1.1 mg/kg single	35.8	41.4	39.2	37	32.3
62	Australia	1.1 mg/kg single	38.4	45.3	43.1	42.9	35.5
63	Australia	1.1 mg/kg single	39.2	48.2	48.5	43	38.1
64	Australia	1.1 mg/kg single	32	32.1	31.6	37.2	34.1
65	Australia	1.1 mg/kg single	32	40.6	36.6	41.8	36.4

## 4 Discussion

The value and usefulness of meta‐analyses of plasma and urine concentrations collected in different horse trials using POP PK have been advocated in the racing industry [11]. The objectives for institutions are to facilitate harmonization in the establishment of international SLs and prescribers and to assist them in determining an individual WT for a horse under their supervision [11]. NLMEM is a potential tool that allows simultaneous analysis of all collected data, old or new, sparse or rich, censored or not, experimental or observational, plasma, urine, or other matrices, where sufficiently large international datasets exist [25]. The NLMEM will estimate central tendencies for key PK parameters such as plasma clearance by estimating “typical values” allowing for optimal summarization of a set of unbalanced data. In a meta‐analysis, a single irrelevant plasma or urine concentration can be comprehensively calculated by optimally and equitably weighing all available data. Metanalytic IPC and IUC can be the basis for risk management by racing organizations in the setting of ISLs. This comprehensive approach is an extension of the current approach, which is based on expert assessment and agreement on the data produced in each jurisdiction.

This study is the first to examine this innovative meta‐analysis approach as proof of concept. Flunixin was selected because it is one of the most widely used NSAIDs in horses [26, 27, 28, 29, 30] and raw data were available from four countries. The current international SLs for flunixin, promulgated by the IFHA, are 1 ng/mL in plasma and 100 ng/mL in urine [13, 14]. Our meta‐analysis aligned with these standards, estimating 1.9 ng/mL for plasma and 70.2 ng/mL for urine. These two values consider only experimental data and ignore the fact that they can be legitimately modified during the risk management step aimed at finding an international consensus. However, our meta‐analysis also suggests that these two current ISLs could be optimized in terms of two factors: the DTs obtained by considering plasma are longer than those resulting from considering urine, and the DT is longer after repeated administration than after a single FM administration. An earlier report by the NLMEM based on 20 horses in Japan identified these two factors [12]. Currently, the matrix of the postrace test depends on the rules of each competition organization, but differences in conclusions can be more closely harmonized through a better alignment of plasma and urine SLs. In particular, plasma and urine sampling in horses is more challenging than in humans; therefore, the possibility of obtaining only one matrix must also be considered. The difference was particularly large after multiple FM administrations, with one horse having a discrepancy of more than 5 days (ID: 20 in Table 6) between the two matrices. The origins of these two issues are easy to identify, and this meta‐analysis makes it possible to propose options that merit exploration by risk managers.

The DT difference between plasma and urine is due to the difference between the experimental urine/plasma ratio estimated at 35.9 in the present meta‐analysis and the regulatory ratio of IFHA, which is operationally equal to 100 (plasma, 1 ng/mL; urine, 100 ng/mL) [12]. These two IFHA values were likely obtained by rounding off the initially calculated values. The second issue of DT being prolonged beyond the 144 h DT [15] for a single IV administration is that the IFHA plasma SL of flunixin (1 ng/mL) has been set throughout the course of the very late terminal phase of flunixin disposition. It should be understood that this late terminal phase is subjected to progressive accumulation when dosing is repeated, with an ineluctable increase in residual plasma concentration during this late phase [31]. This problem can be prevented either by establishing a longer WT after multiple dosing or by possibly increasing the plasma SL to be above this very late terminal phase after reaching steady‐state conditions (about 8–10 days for flunixin).

We explored different scenarios with the aim of minimizing the differences in DT between plasma and urine and between single‐ and multiple‐dose administrations. For example, a scenario in which urinary SL is maintained at 100 ng/mL would require that the plasma ISL be increased to 3 ng/mL to minimize the differences in DTs between plasma and urine. The scenario of IPC (2 ng/mL) still showed DT prolongation after multiple administrations, as 2 ng/mL crossed the very late terminal slope. We also compared the DTs obtained using the current IFHA SL for urine (100 ng/mL) with plasma SLs of 4 and 5 ng/mL retained by HISA [22] and RMTC [23] respectively. With these plasma SLs, multiple doses of FM are no longer associated with prolonged DTs, but the distributions of DTs in the plasma are shifted to the left, resulting in shorter DTs in the plasma than in the urinary SL of IFHA. This is because the operational Rss associated with SLs of 4 and 5 ng/mL, that is, 25 and 20 ng/mL, respectively, were lower than the actual measured Rss of 35.9. According to this analysis, it has been suggested that scenarios of SL 3 ng/mL in plasma and currently 100 ng/mL ISL in urine are effective for avoiding discrepancies between plasma and urine results as well as for preventing the prolongation of DT after multiple administrations.

For risk communication, organizations such as the EHSLC provide DT to help veterinarians estimate future WTs [10]. Because these DTs are based on small samples (6–8 horses), veterinarians must be conservative and set WTs longer than the EHSLC DTs. Statistical analysis recommends doubling the DT from a six‐horse study and multiplying by 1.4 for an eight‐horse study [5]. For flunixin, the suggested DT is 144 h from a trial with four horses, indicating a minimum WT of 288 h (12 days) for a single IV administration. The WT served as the worst‐case scenario, protecting most horses. Our meta‐analysis refined this WT, proposing population WTs of 85 and 192 h (4 and 8 days) for covering 95% of horses after a single IV dose of 1.1 mg/kg or every 12 h five daily IV doses at the same rate. These candidate WTs were significantly shorter than those derived by merely adding a safety factor, particularly in smaller studies [10]. Compared to a previous NLMEM based on 20 Japanese horses, the BSV of plasma clearance increased from 20.8% to 29.8%, and the BSV of steady‐state concentration (Rss) rose from 82.4% to 99.0%, indicating a more conservative model based on international data, but less conservative than that obtained by adding a safety factor [12]. In all cases, the DT after a single 1.1 mg/kg dose was less than 144 h for both plasma and urine with the current ISL [15]. These findings support the 144‐hour DT, suggesting a low risk of positive results at that time. However, as noted earlier, DTs may exceed 144 h in plasma after multiple flunixin doses [12, 32]. While the present study analyzed both single and multiple dosing regimens to evaluate the influence of accumulation on DTs, multiple daily doses of flunixin immediately before racing are highly unlikely, as they would indicate treatment of a clinical condition potentially compromising horse welfare and safety. A single precompetition dose remains the most relevant regulatory scenario, but information on multiple doses provides the supplementary data for prescribing veterinarians for various clinical situations.

An improved approach for prescribing veterinarians would be to estimate the WT for their horses using Bayesian methods. By leveraging a POP PK model, an IBWT can be confidently calculated with only minimal specific data from the horse, such as plasma or urine concentrations [11]. This method also allows for the inclusion of covariates, such as age, breed, and weight, to further refine the estimation. In this meta‐analysis, no demographic covariates were included because of confounding effects, particularly the breed effect, as data for Thoroughbreds are absent in Australia. Despite this limitation, we included standardbred horses because one of the aims of this type of meta‐analysis was to reveal the difficulties and propose solutions. Standardbred data may potentially belong to a different distribution, but because of the limited datasets with confounding variables of breed or geography, it is not possible to determine whether this is the case at this stage. Therefore, further studies with more standardbred data and animals with known levels of fitness from other countries are required to confirm whether the differences between populations are due to the effect of breed. This limitation can be addressed in the future by incorporating new data, at least when sufficient international population data become available. Our POP model (available in Supplemental File S8) can be easily refined in the future by including new data from different breeds from different countries.

The results of the rich scenario show that Bayesian estimates of DTs are close to the true values and are often shorter than those of population‐based WT. In this scenario, differences were observed in cases where the DT was prolonged after multiple administrations in Japan, and these were improved by changing the sampling time from 48 h to 72 h (Supplemental File S9). In addition, the urine DTs of the Bayesian estimates in US horses were shorter than those of the NCA DTs. This is because the urine samples were sparse, leading to a bias with longer observed DTs estimated by the NCA engine than those estimated with more frequent samples. The results obtained clearly show that these Bayesian estimates of DTs are reasonably close to the true values and, for a majority of horses, are significantly shorter than the WT, which, in essence, is a population value aimed at covering the majority of a population of horses with a value that reflects a worst‐case scenario. The reliability of the IBWT depends on the number of blood or urine samples available for the horse being assessed. In this study, only one plasma sample was collected 24 h after either a single IV dose or fifth oral dose, leading to similar results. This meta‐analysis is the first to use a Bayesian approach to predict individual WTs and the results are promising for future research that requires the estimation of WTs in new horses not included in the current model to validate the predictive value of the current POP model.

## 5 Conclusions

Using a POP PK model that aggregated pharmacokinetic (PK) data from 65 horses across four countries, we determined the IPC and IUC of flunixin to be 1.9 and 70.2 ng/mL, respectively. Based on these values, two possible SLs—2 ng/mL in plasma and 70 ng/mL in urine; 3 ng/mL in plasma and 100 ng/mL in urine—were proposed to reduce discrepancies between the plasma and urine results, as well as to shorten DTs following multiple administrations across different countries. The model described in this study can also be used to predict WTs for individual horses using Bayesian estimates, provided at least one plasma and/or urine concentration measurement from that horse is available. While existing non‐POP statistical models remain useful, they may require a more conservative approach to calculating a potential WT compared to Bayesian estimates.

## Conflicts of Interest

The authors declare no conflicts of interest.

## Supporting information


**Data S1:** Results of the NCA (Model 200‐202, linear trapezoidal rule) of the plasma of 65 horses.
**Data S2:** Scatter diagram of two covariates: age (upper) and body weight (BW) (bottom) versus parameters estimated by the NCA engine (clearance (left) and steady‐state distribution volume (central)) and Rss (right) by post hoc values of the NLME model. Visual inspection of these figures suggests that age and BW could be significant covariates, with these parameters decreasing with age and BW.
**Data S3:** Box plots of covariates as countries versus parameters estimated by the NCA engine (clearance [left] and steady‐state distribution volume [central]) and Rss (right) by the post hoc value of the NLME model.
**Data S4:** Plot (lattice by individual) of the dependent variable (plasma [Cobs] and urine [cobsurine] flunixin concentration, red circles) and individual predicted curve (green line) versus time (hours) after dose administration. Plots 1–20: Japanese horses; Plots 21–47: American horses; Plots 48–53: UK horses; Plots 54–65: Australian horses.
**Data S5:** Estimates of individual differences in the plasma and urine DTs of flunixin for different values of the five sets of SLs in plasma and urine. The absolute average of DT differences was 16.0 ± 23.8 h for the current SL (1 vs. 100 ng/mL), 11.9 ±13.9 h for SL 2 vs. 70 ng/mL, 9.8 ±9.1 for SL 3 vs. 100 ng/mL, 10.6 ± 7.1 h for HISA SL (4 vs. 100 ng/mL), and 11.2 ± 7.0 h for RMTC SLs (5 vs. 100 ng/mL).
**Data S6:** Bland–Altman plot to assess the agreement between the two DTs in plasma vs. urine for the current ISL (1 vs. 100 ng/mL), IUC and IPC (2 vs. 70 ng/mL), candidate SL (3 vs. 100 ng/mL), HISA SL in plasma and urine ISL (4 vs. 100 ng/mL), and RMTC SL in plasma and urine ISL (5 vs. 100 ng/mL).
**Data S7:** Comparison of Bayesian estimates between the two scenarios and observed DTs in 65 horses.
**Data S8:** Phoenix code for the final model.
**Data S9:** Comparison of Bayesian estimates using the time points 9, 24, and 48 h or 9, 24, and 72 h versus observed plasma DTs in 10 Japanese horses after multiple administrations.

## Data Availability

The data that support the findings of this study are available from the corresponding author upon reasonable request.
