# Supplementary material for: A Meta‐Analysis of International Flunixin Pharmacokinetics in Horses: Toward Regulatory Harmonization and Individualized Detection Times Using Bayesian Paradigm
Source: Drug Test Anal. 2025 Oct 25;18(1):32–50. doi: 10.1002/dta.3961 (PMC12796560; doi:10.1002/dta.3961)
Supplement: Supplementary file 1 — Data S1: Results of the NCA (Model 200‐202, linear trapezoidal rule) of the plasma of 65 horses. Data S2: Scatter diagram of two covariates: age (upper) and body weight (BW) (bottom) versus parameters estimated by the NCA engine (clearance (left) and steady‐state distribution volume (central)) and Rss (right) by post hoc values of the NLME model. Visual inspection of these figures suggests that age and BW could be significant covariates, with these parameters decreasing with age and BW. Data S3: Box plots of covariates as countries versus parameters estimated by the NCA engine (clearance [left] and steady‐state distribution volume [central]) and Rss (right) by the post hoc value of the NLME model. Data S4: Plot (lattice by individual) of the dependent variable (plasma [Cobs] and urine [cobsurine] flunixin concentration, red circles) and individual predicted curve (green line) versus time (hours) after dose administration. Plots 1–20: Japanese horses; Plots 21–47: American horses; Plots 48–53: UK horses; Plots 54–65: Australian horses. Data S5: Estimates of individual differences in the plasma and urine DTs of flunixin for different values of the five sets of SLs in plasma and urine. The absolute average of DT differences was 16.0 ± 23.8 h for the current SL (1 vs. 100 ng/mL), 11.9 ±13.9 h for SL 2 vs. 70 ng/mL, 9.8 ±9.1 for SL 3 vs. 100 ng/mL, 10.6 ± 7.1 h for HISA SL (4 vs. 100 ng/mL), and 11.2 ± 7.0 h for RMTC SLs (5 vs. 100 ng/mL). Data S6: Bland–Altman plot to assess the agreement between the two DTs in plasma vs. urine for the current ISL (1 vs. 100 ng/mL), IUC and IPC (2 vs. 70 ng/mL), candidate SL (3 vs. 100 ng/mL), HISA SL in plasma and urine ISL (4 vs. 100 ng/mL), and RMTC SL in plasma and urine ISL (5 vs. 100 ng/mL). Data S7: Comparison of Bayesian estimates between the two scenarios and observed DTs in 65 horses. Data S8: Phoenix code for the final model. Data S9: Comparison of Bayesian estimates using the time points 9, 24, and 48 h or 9, 24, and 72 h ve [file DTA-18-32-s001.docx]

Supplemental File 1: Results of the NCA (Model 200-202, linear trapezoidal rule) of the plasma of 65 horses.

|  | Dose | Half-life | C0 | AUC  (0-Tlast) | AUC  (0-inf) | Clearance | Vss |
| --- | --- | --- | --- | --- | --- | --- | --- |
| Hors/Units | mg/kg | h | µg/mL | ng*h/mL | ng*h/mL | mL/kg/h | mL/kg |
| 1 | 1.10 | 90.0 | 12.45 | 33720 | 33733 | 32.6 | 136 |
| 2 | 1.10 | 26.9 | 13.23 | 29831 | 29837 | 36.9 | 120 |
| 3 | 1.10 | 13.7 | 11.45 | 27597 | 27601 | 39.9 | 150 |
| 4 | 1.10 | 15.5 | 7.18 | 24999 | 25002 | 44 | 138 |
| 5 | 1.10 | 16.0 | 9.64 | 25936 | 25939 | 42.4 | 142 |
| 6 | 1.10 | 15.6 | 5.95 | 21025 | 21033 | 52.3 | 204 |
| 7 | 1.10 | 28.2 | 14.86 | 26821 | 26832 | 41 | 106 |
| 8 | 1.10 | 16.1 | 7.13 | 17447 | 17450 | 63 | 195 |
| 9 | 1.10 | 35.2 | 6.98 | 28921 | 28929 | 38 | 170 |
| 10 | 1.10 | 53.1 | 10.72 | 32097 | 32140 | 34.2 | 150 |
| 11 | 1.10 | 49.3 | 11.00 | 28053 | 28062 | 39.2 | 151 |
| 12 | 1.10 | 41.1 | 11.85 | 23010 | 23017 | 47.8 | 150 |
| 13 | 1.10 | 37.0 | 7.16 | 25487 | 25496 | 43.1 | 192 |
| 14 | 1.10 | 44.1 | 12.15 | 46415 | 46436 | 23.7 | 143 |
| 15 | 1.10 | 105.0 | 4.67 | 25903 | 25946 | 42.4 | 226 |
| 16 | 1.10 | 32.6 | 7.50 | 21301 | 21308 | 51.6 | 263 |
| 17 | 1.10 | 60.7 | 10.00 | 37636 | 37674 | 29.2 | 180 |
| 18 | 1.10 | 36.5 | 6.21 | 17550 | 17557 | 62.7 | 250 |
| 19 | 1.10 | 34.1 | 9.59 | 24465 | 24470 | 45 | 218 |
| 20 | 1.10 | 60.3 | 11.37 | 40322 | 40393 | 27.2 | 167 |
| 21 | 1.10 | 4.8 | 13.96 | 21021 | 21035 | 52.3 | 139 |
| 22 | 1.10 | 3.4 | 14.00 | 27803 | 27811 | 39.6 | 115 |
| 23 | 1.10 | 4.1 | 11.76 | 25776 | 25783 | 42.7 | 127 |
| 24 | 1.10 | 3.4 | 14.98 | 22936 | 22944 | 47.9 | 132 |
| 25 | 1.10 | 6.0 | 13.60 | 25910 | 25928 | 42.4 | 145 |
| 26 | 1.10 | 4.6 | 13.54 | 24346 | 24356 | 45.2 | 124 |
| 27 | 1.10 | 4.3 | 12.23 | 23336 | 23347 | 47.1 | 144 |
| 28 | 1.10 | 4.0 | 13.43 | 20568 | 20576 | 53.5 | 150 |
| 29 | 1.10 | 4.2 | 12.55 | 20666 | 20673 | 53.2 | 155 |
| 30 | 1.10 | 4.2 | 11.75 | 25250 | 25259 | 43.5 | 155 |
| 31 | 1.10 | 4.1 | 15.46 | 27397 | 27414 | 40.1 | 129 |
| 32 | 1.10 | 4.1 | 13.56 | 24408 | 24419 | 45 | 128 |
| 33 | 1.10 | 4.9 | 13.28 | 30222 | 30233 | 36.4 | 128 |
| 34 | 1.10 | 4.4 | 14.14 | 27133 | 27146 | 40.5 | 120 |
| 35 | 1.10 | 3.7 | 13.20 | 27761 | 27777 | 39.6 | 133 |
| 36 | 1.10 | 4.7 | 13.64 | 20041 | 20049 | 54.9 | 135 |
| 37 | 0.90 | 3.7 | 12.22 | 22242 | 22250 | 40.3 | 145 |
| 38 | 0.92 | 10.7 | 11.63 | 22883 | 22899 | 40.3 | 154 |
| 39 | 0.91 | 6.8 | 14.93 | 25378 | 25401 | 35.7 | 150 |
| 40 | 0.84 | 8.0 | 11.05 | 30934 | 30950 | 27.3 | 135 |
| 41 | 0.88 | 19.6 | 13.84 | 18397 | 18427 | 47.8 | 144 |
| 42 | 0.91 | 6.0 | 13.87 | 26532 | 26554 | 34.3 | 127 |
| 43 | 1.07 | 6.9 | 15.45 | 26876 | 26888 | 39.7 | 129 |
| 44 | 1.01 | 3.9 | 14.38 | 27992 | 28000 | 36.2 | 129 |
| 45 | 0.91 | 4.3 | 11.53 | 29608 | 29620 | 30.7 | 135 |
| 46 | 0.92 | 4.0 | 14.71 | 19925 | 19936 | 46.3 | 123 |
| 47 | 0.96 | 4.9 | 10.90 | 20831 | 20839 | 46.2 | 156 |
| 48 | 1.10 | 28.7 | 12.09 | 32989 | 32997 | 33.3 | 122 |
| 49 | 1.10 | 24.3 | 8.79 | 34486 | 34490 | 31.9 | 159 |
| 50 | 1.10 | 25.8 | 11.83 | 37983 | 37997 | 28.9 | 117 |
| 51 | 1.10 | 28.4 | 9.61 | 30630 | 30635 | 35.9 | 143 |
| 52 | 1.10 | 22.1 | 8.73 | 20259 | 20262 | 54.3 | 158 |
| 53 | 1.10 | 29.9 | 12.00 | 38256 | 38262 | 28.7 | 115 |
| 54 | 1.10 | 4.0 | 13.36 | 15240 | 15257 | 72.1 | 199 |
| 55 | 1.10 | 4.8 | 14.43 | 14161 | 14175 | 77.6 | 166 |
| 56 | 1.10 | 4.1 | 13.31 | 14449 | 14461 | 76.1 | 180 |
| 57 | 1.10 | 4.0 | 10.89 | 10416 | 10422 | 105.5 | 179 |
| 58 | 1.10 | 3.6 | 12.08 | 22252 | 22304 | 49.3 | 204 |
| 59 | 1.10 | 3.2 | 8.30 | 15316 | 15321 | 71.8 | 185 |
| 60 | 1.10 | 3.9 | 14.33 | 15501 | 15512 | 70.9 | 179 |
| 61 | 1.10 | 3.8 | 10.29 | 16891 | 16902 | 65.1 | 174 |
| 62 | 1.10 | 4.0 | 14.89 | 18160 | 18177 | 60.5 | 168 |
| 63 | 1.10 | 3.7 | 10.05 | 21133 | 21155 | 52 | 172 |
| 64 | 1.10 | 3.9 | 14.96 | 11867 | 11872 | 92.7 | 189 |
| 65 | 1.10 | 3.3 | 10.73 | 13090 | 13094 | 84 | 206 |

*AUClast: Area Under the Curve up to the last measured concentration (no extrapolation to infinity); AUClast_Dose is the AUC scaled by dose unit. Vss; Steady-state volume of distribution*

Supplemental File 2: Scatter diagram of two covariates: age (upper) and body weight (BW) (bottom) versus parameters estimated by the NCA engine (clearance (left) and steady-state distribution volume (central)) and Rss (right) by post hoc values of the NLME model. Visual inspection of these figures suggests that age and BW could be significant covariates, with these parameters decreasing with age and BW.
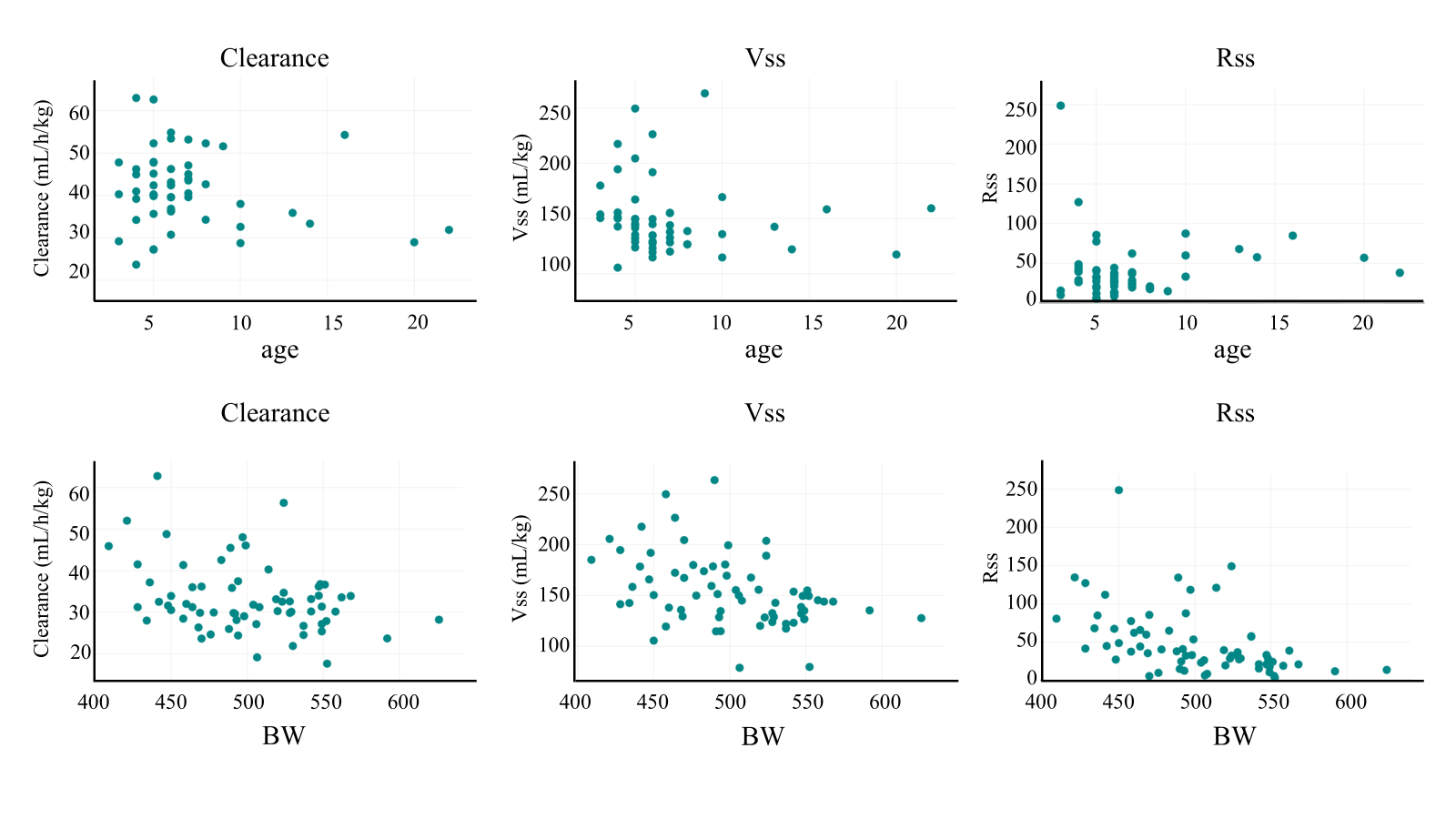


Supplemental File 3: Box Plots of covariates as countries versus parameters estimated by the NCA engine (clearance (left) and steady-state distribution volume (central)) and Rss (right) by the post hoc value of the NLME model.


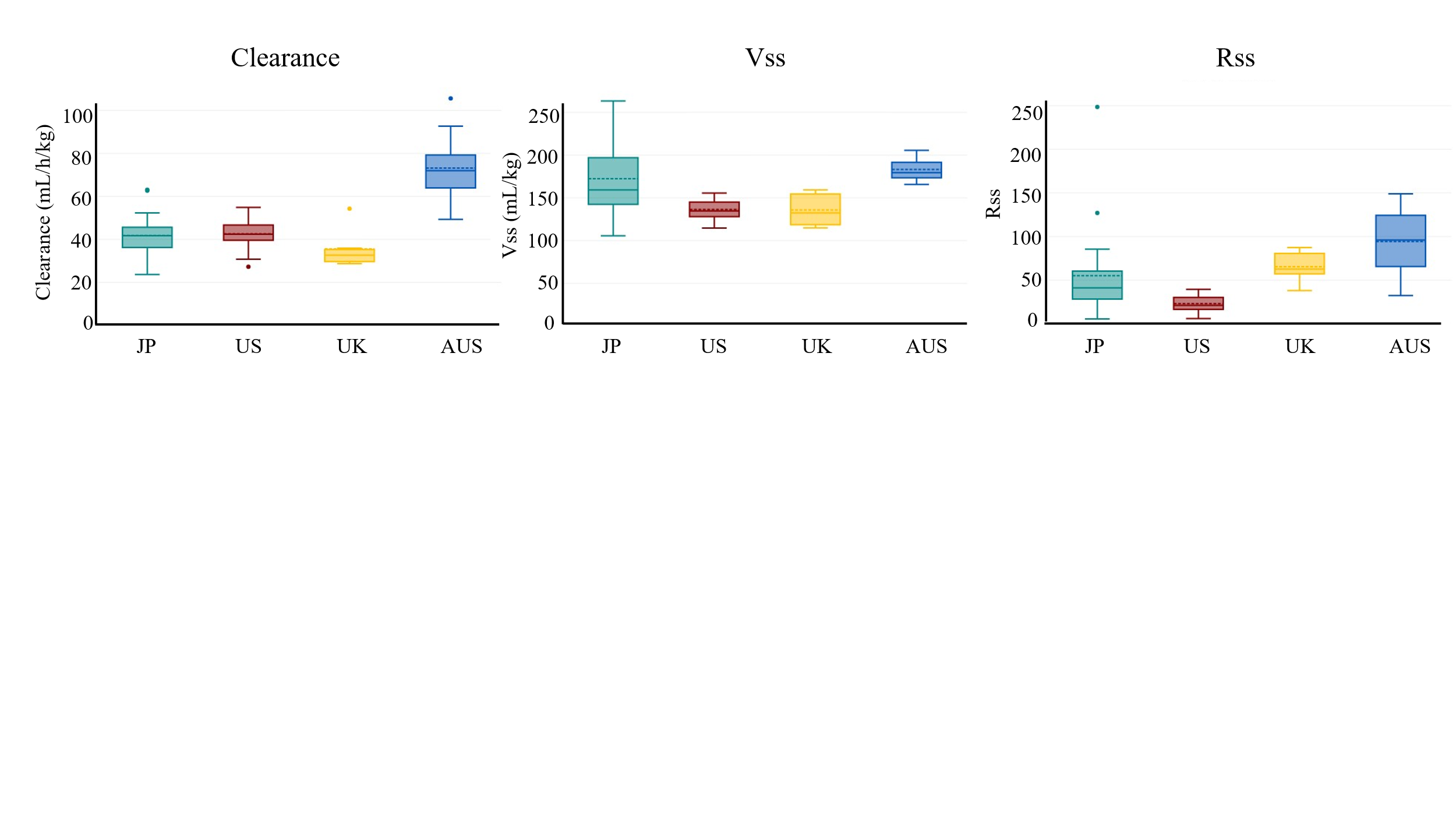


Supplemental File 4: Plot (lattice by individual) of the dependent variable (plasma (Cobs) and urine (cobsurine) flunixin concentration, red circles) and individual predicted curve (green line) vs. time (hours) after dose administration. Plots1–20: Japanese horses; Plots 21–47: American horses; Plots 48–53: UK horses; Plots 54–65: Australian horses.

Supplemental File 5: Estimates of individual differences in the plasma and urine DTs of flunixin for different values of the five sets of SLs in plasma and urine. The absolute average of DT differences was 16.0 ± 23.8 h for the current SL (1 vs 100 ng/mL), 11.9 ±13.9 h for SL 2 vs 70 ng/mL, 9.8 ±9.1 for SL 3 vs 100 ng/mL, 10.6 ± 7.1 h for HISA SL (4 vs 100 ng/mL), and 11.2 ± 7.0 h for RMTC SLs (5 vs 100 ng/mL).

|  |  | |  | | **Difference for DT (h) between plasma and urine for 5 sets of SL** | | | | |
| --- | --- | --- | --- | --- | --- | --- | --- | --- | --- |
| **ID** | | **nation** | | **dosing** | **1 vs 100** | **2 vs 70** | **3 vs 100** | **4 vs 100** | **5 vs 100** |
| 1 | | Japan | | 1.1 mg/kg single | 4.4 | -0.9 | -1.8 | -3.2 | -4.5 |
| 2 | | Japan | | 1.1 mg/kg single | 2.4 | -1.2 | -2.1 | -4.4 | -6.6 |
| 3 | | Japan | | 1.1 mg/kg single | 11.1 | -2.8 | -1.8 | -8.2 | -11.5 |
| 4 | | Japan | | 1.1 mg/kg single | 12.5 | -3.2 | -4.9 | -10.0 | -15.0 |
| 5 | | Japan | | 1.1 mg/kg single | 16.0 | -0.4 | -1.5 | -3.5 | -5.5 |
| 6 | | Japan | | 1.1 mg/kg single | 13.4 | -0.7 | -4.2 | -8.3 | -12.5 |
| 7 | | Japan | | 1.1 mg/kg single | 6.8 | -9.0 | -11.1 | -12.0 | -12.8 |
| 8 | | Japan | | 1.1 mg/kg single | -3.9 | -19.6 | -17.7 | -21.3 | -22.5 |
| 9 | | Japan | | 1.1 mg/kg single | 12.5 | -0.2 | -3.1 | -10.9 | -12.0 |
| 10 | | Japan | | 1.1 mg/kg single | NC | NC | NC | NC | NC |
| 11 | | Japan | | 1.1 mg/kg q 24 h 5 days | 25.1 | -0.4 | -2.3 | -5.1 | -7.8 |
| 12 | | Japan | | 1.1 mg/kg q 24 h 5 days | 6.3 | -20.9 | -20.2 | -26.8 | -31.1 |
| 13 | | Japan | | 1.1 mg/kg q 24 h 5 days | 42.3 | 18.9 | 8.5 | 0.5 | -1.2 |
| 14 | | Japan | | 1.1 mg/kg q 24 h 5 days | 72.8 | 7.7 | 30.5 | 4.1 | -4.4 |
| 15 | | Japan | | 1.1 mg/kg q 24 h 5 days | 31.2 | -3.2 | -5.1 | -6.5 | -7.9 |
| 16 | | Japan | | 1.1 mg/kg q 24 h 5 days | 87.8 | 44.7 | 36.1 | 3.3 | 2.4 |
| 17 | | Japan | | 1.1 mg/kg q 24 h 5 days | 89.2 | 35.6 | 22.9 | 19.2 | 15.5 |
| 18 | | Japan | | 1.1 mg/kg q 24 h 5 days | 26.4 | -35.8 | -3.3 | -6.3 | -9.3 |
| 19 | | Japan | | 1.1 mg/kg q 24 h 5 days | 40.5 | 22.3 | 16.2 | 9.7 | 3.2 |
| 20 | | Japan | | 1.1 mg/kg q 24 h 5 days | 123.5 | 84.2 | 47.2 | 39.0 | 30.7 |
| 21 | | US | | 1.1mg/kg single | 1.0 | -6.8 | -5.8 | -7.9 | -9.8 |
| 22 | | US | | 1.1mg/kg single | -1.7 | -0.5 | -8.5 | -11.1 | -13.5 |
| 23 | | US | | 1.1mg/kg single | 2.3 | -2.3 | -6.0 | -7.9 | -9.7 |
| 24 | | US | | 1.1mg/kg single | -3.9 | -11.2 | -12.8 | -14.7 | -15.6 |
| 25 | | US | | 1.1mg/kg single | 13.9 | 5.0 | 5.8 | 3.0 | 0.1 |
| 26 | | US | | 1.1mg/kg single | -4.3 | -10.0 | -11.2 | -13.3 | -14.3 |
| 27 | | US | | 1.1mg/kg single | -5.3 | -10.5 | -11.4 | -13.5 | -15.3 |
| 28 | | US | | 1.1mg/kg single | -3.7 | -9.1 | -9.1 | -11.3 | -12.3 |
| 29 | | US | | 1.1mg/kg single | -1.4 | -7.6 | -10.4 | -12.2 | -14.0 |
| 30 | | US | | 1.1mg/kg single | 1.0 | -0.8 | -4.4 | -9.0 | -10.1 |
| 31 | | US | | 1.1mg/kg single | -3.4 | -8.6 | -9.4 | -10.9 | -12.4 |
| 32 | | US | | 1.1mg/kg single | -4.0 | -9.2 | -10.1 | -12.8 | -13.6 |
| 33 | | US | | 1.1mg/kg single | 4.9 | 0.9 | -1.5 | -5.1 | -8.1 |
| 34 | | US | | 500 mg/horse single | -1.2 | -7.6 | -7.4 | -9.2 | -11.1 |
| 35 | | US | | 500 mg/horse single | -5.7 | -10.0 | -11.5 | -11.8 | -13.7 |
| 36 | | US | | 500 mg/horse single | -2.4 | -9.4 | -9.8 | -11.2 | -12.6 |
| 37 | | US | | 500 mg/horse single | -0.2 | -1.0 | -4.9 | -8.6 | -9.9 |
| 38 | | US | | 500 mg/horse single | 15.8 | 3.4 | -0.2 | -4.5 | -8.5 |
| 39 | | US | | 500 mg/horse single | 16.3 | 6.9 | 6.6 | 4.1 | 1.7 |
| 40 | | US | | 500 mg/horse single | 17.3 | 10.0 | 5.9 | 1.4 | -0.2 |
| 41 | | US | | 500 mg/horse single | NC | NC | NC | NC | NC |
| 42 | | US | | 500 mg/horse single | 8.1 | 0.7 | 0.3 | -2.8 | -5.8 |
| 43 | | US | | 500 mg/horse single | 2.6 | -4.0 | -9.2 | -11.6 | -13.1 |
| 44 | | US | | 500 mg/horse single | 2.5 | 0.6 | -2.3 | -6.7 | -7.7 |
| 45 | | US | | 500 mg/horse single | 4.4 | 1.8 | 0.4 | -2.1 | -4.6 |
| 46 | | US | | 500 mg/horse single | -2.2 | -8.0 | -8.2 | -10.8 | -11.9 |
| 47 | | US | | 500 mg/horse single | -0.4 | -5.0 | -10.6 | -11.8 | -13.0 |
| 48 | | UK | | 1.1mg/kg single | 31.7 | -16.2 | -3.8 | -5.3 | -6.8 |
| 49 | | UK | | 1.1mg/kg single | 37.2 | -7.2 | -1.2 | -3.8 | -6.3 |
| 50 | | UK | | 1.1mg/kg single | 41.2 | -14.3 | -1.5 | -3.3 | -4.4 |
| 51 | | UK | | 1.1mg/kg single | 10.2 | -18.8 | -13.3 | -15.8 | -18.3 |
| 52 | | UK | | 1.1mg/kg single | 1.3 | -14.6 | -11.2 | -15.4 | -16.4 |
| 53 | | UK | | 1.1mg/kg single | 10.2 | -26.6 | -16.9 | -18.6 | -20.4 |
| 54 | | Australia | | 1.1mg/kg single | 0.7 | -6.0 | -5.6 | -6.6 | -7.6 |
| 55 | | Australia | | 1.1mg/kg single | 3.1 | -5.5 | -3.5 | -5.5 | -7.5 |
| 56 | | Australia | | 1.1mg/kg single | -19.9 | -34.4 | -25.4 | -27.4 | -29.4 |
| 57 | | Australia | | 1.1mg/kg single | -3.7 | -12.9 | -10.4 | -11.1 | -11.8 |
| 58 | | Australia | | 1.1mg/kg single | -9.3 | -20.8 | -15.0 | -21.0 | -21.0 |
| 59 | | Australia | | 1.1mg/kg single | -3.3 | -8.4 | -7.3 | -9.3 | -11.3 |
| 60 | | Australia | | 1.1mg/kg single | -5.3 | -11.3 | -10.6 | -12.2 | -13.8 |
| 61 | | Australia | | 1.1mg/kg single | -1.2 | -7.6 | -6.1 | -7.3 | -8.4 |
| 62 | | Australia | | 1.1mg/kg single | -4.5 | -9.1 | -10.9 | -11.7 | -12.5 |
| 63 | | Australia | | 1.1mg/kg single | -3.7 | -9.8 | -9.6 | -11.0 | -11.6 |
| 64 | | Australia | | 1.1mg/kg single | -5.2 | -14.2 | -13.2 | -13.6 | -14.0 |
| 65 | | Australia | | 1.1mg/kg single | -9.8 | -12.6 | -1.5 | -13.3 | -14.4 |

Supplemental File 6: Bland-Altman plot to assess the agreement between the two DTs in plasma vs. urine for the current ISL (1 vs. 100 ng/mL), IUC and IPC (2 vs. 70 ng/mL), candidate SL (3 vs. 100 ng/mL), HISA SL in plasma and urine ISL (4 vs. 100 ng/mL), and RMTC SL in plasma and urine ISL (5 vs. 100 ng/mL).


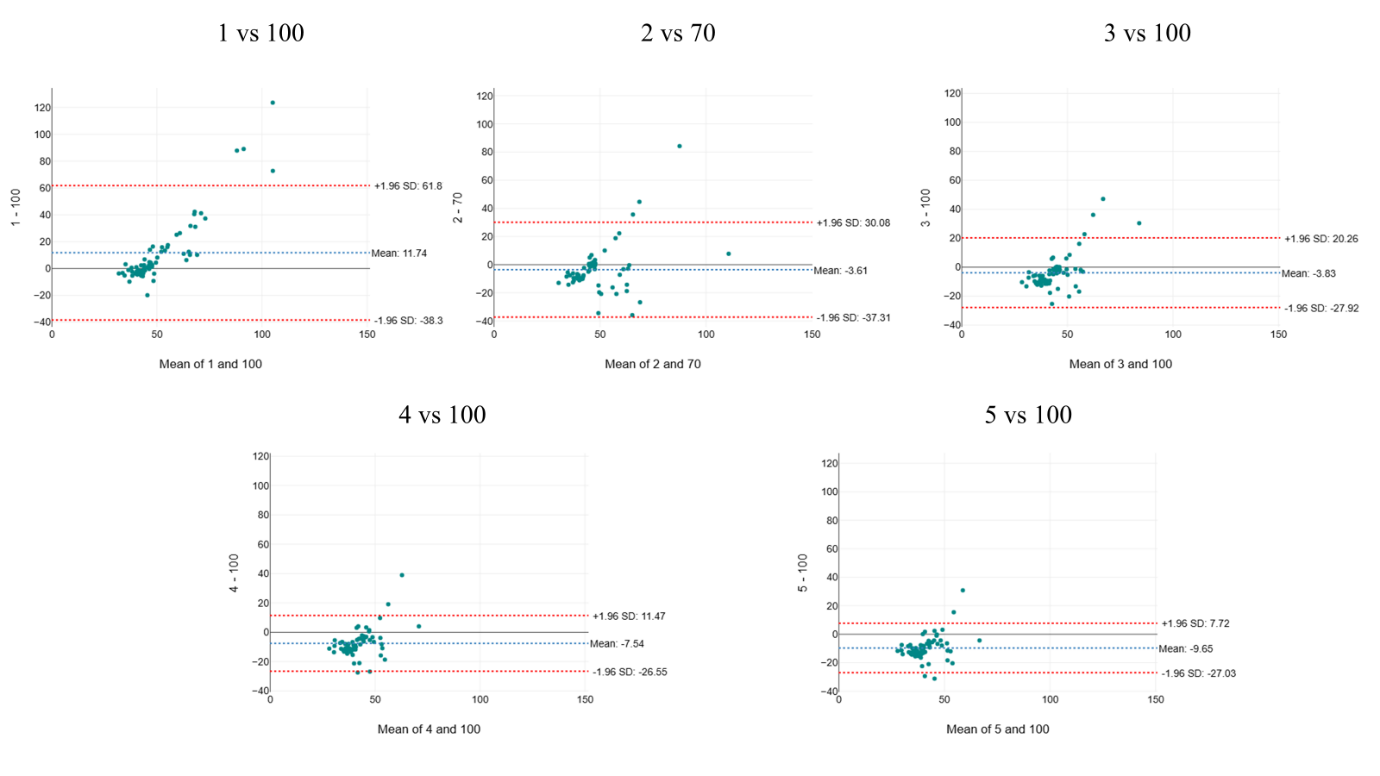


Supplemental File 7: Comparison of Bayesian estimates between the two scenarios and observed DTs in 65 horses.

|  |  |  | Plasma SL: 1 ng/mL | | |  |  |
| --- | --- | --- | --- | --- | --- | --- | --- |
| ID | Nation | Dosing | observed | Bayesian Sparse | Bayesian  Rich | Bias for 1 sample | Bias for 4 samples |
|  |  |  |  | n= 1 | n=4 | h | h |
|  |  |  |  | sample | samples |  |  |
| 1 | Japan | 1.1 mg/kg single | 51.7 | 60.3 | 53.1 | -8.6 | -1.4 |
| 2 | Japan | 1.1 mg/kg single | 47.4 | 46.6 | 44.8 | 0.8 | 2.6 |
| 3 | Japan | 1.1 mg/kg single | 68.2 | 58.1 | 63.9 | 10.1 | 4.3 |
| 4 | Japan | 1.1 mg/kg single | 58.3 | 46.4 | 61.0 | 11.9 | -2.7 |
| 5 | Japan | 1.1 mg/kg single | 62.9 | 50.0 | 63.4 | 12.9 | -0.5 |
| 6 | Japan | 1.1 mg/kg single | 60.5 | 48.6 | 65.4 | 11.9 | -4.9 |
| 7 | Japan | 1.1 mg/kg single | 47.5 | 42.7 | 45.8 | 4.8 | 1.7 |
| 8 | Japan | 1.1 mg/kg single | 46.7 | 42.2 | 43.2 | 4.5 | 3.5 |
| 9 | Japan | 1.1 mg/kg single | 71.4 | 66.3 | 101.8 | 5.1 | -30.4 |
| 10 | Japan | 1.1 mg/kg single | 104.2 | 53.4 | 72.7 | 50.8 | 31.5 |
| 11 | Japan | 1.1 mg/kg q 24 h 5 days | 71.9 | 68.4 | 65.1 | 3.5 | 6.8 |
| 12 | Japan | 1.1 mg/kg q 24 h 5 days | 67.1 | 71.2 | 75.4 | -4.1 | -8.3 |
| 13 | Japan | 1.1 mg/kg q 24 h 5 days | 89.1 | 90.1 | 98.2 | -1.0 | -9.1 |
| 14 | Japan | 1.1 mg/kg q 24 h 5 days | 141.5 | 150.4 | 113.8 | -8.9 | 27.7 |
| 15 | Japan | 1.1 mg/kg q 24 h 5 days | 83.8 | 94.3 | 84.5 | -10.5 | -0.7 |
| 16 | Japan | 1.1 mg/kg q 24 h 5 days | 132 | 83.4 | 96.3 | 48.6 | 35.7 |
| 17 | Japan | 1.1 mg/kg q 24 h 5 days | 135.8 | 109.7 | 133.7 | 26.1 | 2.1 |
| 18 | Japan | 1.1 mg/kg q 24 h 5 days | 74.1 | 74.1 | 70.7 | 0.0 | 3.4 |
| 19 | Japan | 1.1 mg/kg q 24 h 5 days | 88 | 103.4 | 112.0 | -15.4 | -24.0 |
| 20 | Japan | 1.1 mg/kg q 24 h 5 days | **166.9** | 113.7 | 99.3 | 53.2 | 67.6 |
| 21 | US | 1.1mg/kg single | 40.7 | 42.6 | 42.9 | -1.9 | -2.2 |
| 22 | US | 1.1mg/kg single | 41.7 | 44.4 | 62.1 | -2.7 | -20.4 |
| 23 | US | 1.1mg/kg single | 43.6 | 46.2 | 50.6 | -2.6 | -7.0 |
| 24 | US | 1.1mg/kg single | 40.1 | 42.2 | 59.0 | -2.1 | -18.9 |
| 25 | US | 1.1mg/kg single | 53.6 | 55.6 | 64.4 | -2.0 | -10.8 |
| 26 | US | 1.1mg/kg single | 38.5 | 41.3 | 43.1 | -2.8 | -4.6 |
| 27 | US | 1.1mg/kg single | 39.8 | 45.0 | 46.6 | -5.2 | -6.8 |
| 28 | US | 1.1mg/kg single | 37.9 | 43.1 | 43.8 | -5.2 | -5.9 |
| 29 | US | 1.1mg/kg single | 43.6 | 57.7 | 50.7 | -14.1 | -7.1 |
| 30 | US | 1.1mg/kg single | 46.1 | 53.8 | 53.8 | -7.7 | -7.7 |
| 31 | US | 1.1mg/kg single | 41.8 | 50.8 | 52.7 | -9.0 | -10.9 |
| 32 | US | 1.1mg/kg single | 38.8 | 42.9 | 44.7 | -4.1 | -5.9 |
| 33 | US | 1.1mg/kg single | 48.8 | 56.0 | 55.2 | -7.2 | -6.4 |
| 34 | US | 500 mg/horse single | 40.5 | 44.8 | 45.7 | -4.3 | -5.2 |
| 35 | US | 500 mg/horse single | 40.4 | 48.6 | 50.5 | -8.2 | -10.1 |
| 36 | US | 500 mg/horse single | 37 | 38.8 | 39.2 | -1.8 | -2.2 |
| 37 | US | 500 mg/horse single | 43.8 | 50.3 | 53.7 | -6.5 | -9.9 |
| 38 | US | 500 mg/horse single | 60.3 | 55.2 | 65.6 | 5.1 | -5.3 |
| 39 | US | 500 mg/horse single | 56.2 | 60.0 | 63.5 | -3.8 | -7.3 |
| 40 | US | 500 mg/horse single | 63.9 | 71.5 | 75.9 | -7.6 | -12.0 |
| 41 | US | 500 mg/horse single | 61.5 | 42.6 | 60.7 | 18.9 | 0.8 |
| 42 | US | 500 mg/horse single | 54.2 | 56.1 | 69.3 | -1.9 | -15.1 |
| 43 | US | 500 mg/horse single | 49 | 51.0 | 51.2 | -2.0 | -2.2 |
| 44 | US | 500 mg/horse single | 45.1 | 53.1 | 54.5 | -8.0 | -9.4 |
| 45 | US | 500 mg/horse single | 49.2 | 62.1 | 59.6 | -12.9 | -10.4 |
| 46 | US | 500 mg/horse single | 39.2 | 44.2 | 43.5 | -5.0 | -4.3 |
| 47 | US | 500 mg/horse single | 46.1 | 50.1 | 48.8 | -4.0 | -2.7 |
| 48 | UK | 1.1mg/kg single | 81.8 | 55.2 | 66.1 | 26.6 | 15.7 |
| 49 | UK | 1.1mg/kg single | 91.6 | 69.1 | 67.9 | 22.5 | 23.7 |
| 50 | UK | 1.1mg/kg single | 91.6 | 56.4 | 84.6 | 35.2 | 7.0 |
| 51 | UK | 1.1mg/kg single | 70.8 | 60.1 | 78.9 | 10.7 | -8.1 |
| 52 | UK | 1.1mg/kg single | 48.3 | 44.8 | 46.5 | 3.5 | 1.8 |
| 53 | UK | 1.1mg/kg single | 74.2 | 61.0 | 71.6 | 13.2 | 2.6 |
| 54 | Australia | 1.1mg/kg single | 38.2 | 43.1 | 41.7 | -4.9 | -3.5 |
| 55 | Australia | 1.1mg/kg single | 36.6 | 37.9 | 36.8 | -1.3 | -0.2 |
| 56 | Australia | 1.1mg/kg single | 35.5 | 39.2 | 38.7 | -3.7 | -3.2 |
| 57 | Australia | 1.1mg/kg single | 30 | 29.1 | 29.7 | 0.9 | 0.3 |
| 58 | Australia | 1.1mg/kg single | 43.7 | 76.8 | 68.9 | -33.1 | -25.2 |
| 59 | Australia | 1.1mg/kg single | 32 | 35.6 | 33.8 | -3.6 | -1.8 |
| 60 | Australia | 1.1mg/kg single | 35.6 | 39.6 | 38.6 | -4.0 | -3.0 |
| 61 | Australia | 1.1mg/kg single | 35.8 | 41.4 | 39.2 | -5.6 | -3.4 |
| 62 | Australia | 1.1mg/kg single | 38.4 | 45.3 | 43.1 | -6.9 | -4.7 |
| 63 | Australia | 1.1mg/kg single | 39.2 | 48.2 | 48.5 | -9.0 | -9.3 |
| 64 | Australia | 1.1mg/kg single | 32 | 32.1 | 31.6 | -0.1 | 0.4 |
| 65 | Australia | 1.1mg/kg single | 32 | 40.6 | 36.6 | -8.6 | -4.6 |

Supplemental File 8: Phoenix code for the final model.

####### Phoenix CODE developed by Taisuke Kuroda 07/2025 ##############

#Green sentences are comments. All comments start with an #

test(){

#equation for the IV disposition for 3-cp model

deriv(A1 = - Cl * C - Cl2 * (C - C2) - Cl3 * (C - C3))

deriv(A2 = Cl2 * (C - C2))

deriv(A3 = Cl3 * (C - C3))

dosepoint(A1)

C = A1 / V

C2 = A2 / V2

C3 = A3 / V3

Curine=Rss*C

error(CEps = 0.116665847216959)

error(CEpsurine = 0.10561216446523)

#observed IV plasma concentration with the additive+multiplicative model; BQL given as a static concentration

observe(CObs =C + CEps * sqrt(1 + C^2 * (CMultStdev/sigma())^2),bql=0.1)

#observed IV urine concentration with the additive+multiplicative model; BQL given as a static concentration

observe(CObsurine =Curine + CEpsurine * sqrt(1 + Curine^2 * (CMultStdevurine/sigma())^2),bql=1)

stparm(V = tvV * exp(nV))

stparm(V2 = tvV2 * exp(nV2))

stparm(V3 = tvV3 * exp(nV3))

stparm(Cl = tvCl * exp(nCl))

stparm(Cl2 = tvCl2 * exp(nC12) )

stparm(Cl3 = tvCl3 * exp(nC13) )

stparm(Rss = tvRss * exp(nRss) )

stparm(CMultStdev = tvCMultStdev)

stparm(CMultStdevurine = tvCMultStdevurine)

fixef(tvV = c(,121.575949369328,))

fixef(tvV2 = c(,8.82707919121307,))

fixef(tvV3 = c(,31.6646844234648,))

fixef(tvCl = c(,47.5685590900195,))

fixef(tvCl2 = c(,0.217291123177233,))

fixef(tvCl3 = c(,7.64122776877917,))

fixef(tvRss = c(,36.6709755851391,))

fixef(tvCMultStdev = c(,0.153233124116505,))

fixef(tvCMultStdevurine = c(,0.438616381252576,))

# block replace diag to get a full OMEGA matrix

ranef(block(nV, nCl,nC12,nC13 ,nV2,nV3,nRss) = c(0.032195696,0.030017194,0.085002354,-0.016371482,0.050308178,0.30297991,-0.027264179,0.029621872,0.23711958,0.35182061,0.0474638,0.050918102,0.18063373,0.049174828,0.33517766,0.011125665,0.032441246,0.11668592,0.1900967,0.070160742,0.13975201,0.083393209,0.15078812,0.029892009,-0.035471922,0.097778827,0.00010347813,0.68318634))

#secondary parameters

secondary(tvVss=tvV+tvV2+tvV3)

secondary(Ke = tvCl/tvV)

secondary(K12 = tvCl2/tvV)

secondary(K21 = tvCl2/tvV2)

secondary(K13 = tvCl3/tvV)

secondary(K31 = tvCl3/tvV3)

secondary(tvKe=tvCl/tvV)

secondary(tvK12=tvCl2/tvV)

secondary(tvK13=tvCl3/tvV)

secondary(tvK21=tvCl2/tvV2)

secondary(tvK31=tvCl3/tvV3)

secondary(tvMRTIV=tvVss/tvCl)

secondary(a0=tvKe*tvK21*tvK31)

secondary(a1=tvKe*tvK31+tvK21*tvK31+tvK21*tvK13+tvKe*tvK21+tvK31*tvK12)secondary(a2=tvKe+tvK12+tvK13+tvK21+tvK31)

secondary(p=a1-a2^2/3)

secondary(q=2*a2^3/27-a1*a2/3+a0)

secondary(r1=(-(p^3/27))^0.5)

secondary(r2=2*r1^0.3333)

secondary(PHI=acos(-q/(2*r1))/3)

secondary(root1=-(cos(PHI)*r2-(a2/3)))

secondary(root2=-(cos(PHI+2*3.14159/3)*r2-a2/3))

secondary(root3=-(cos(PHI+4*3.14159/3)*r2-a2/3))

root1

root2

root3

#to compute Alpha>Beta>gamma otherwise A, B and C are false

secondary(tvAlpha=root1>root2 &&root1>root3 && root2>root3?root1:root2)

secondary(tvBeta=root1>root2 &&root1>root3 && root2>root3?root2:root3)

secondary(tvGamma=root1>root2 &&root1>root3 && root2>root3?root3:root1)

secondary(tvHL_alpha=ln(2)/tvAlpha)

secondary(tvHL_Beta=ln(2)/tvBeta)

secondary(tvHL_Gamma=ln(2)/tvGamma)

#to edit to name alpha>beta>gamma to compute consistently A, B and G

#for a dose of 110000ng/kg : to edit for another dose

Dose=110000

secondary(tvA=((Dose)/tvV)*((tvK21-tvAlpha)/(tvAlpha-tvBeta))*(tvK31-tvAlpha)/(tvAlpha-tvGamma))

secondary(tvB=((Dose)/tvV)*((tvK21-tvBeta)/(tvBeta-tvAlpha))*(tvK31-tvBeta)/(tvBeta-tvGamma))

secondary(tvC=((Dose)/tvV)*((tvK21-tvGamma)/(tvGamma-tvBeta))*(tvK31-tvGamma)/(tvGamma-tvAlpha))

#Estimate of Toutain model approach

secondary(EPC=1100000/(tvCl*24))

secondary(IPC=EPC/500)

secondary(IUC=IPC*tvRss)

}

Supplemental File 9: Comparison of Bayesian estimates using the time points 9, 24, and 48 h or 9, 24, and 72 h versus observed plasma DTs in 10 Japanese horses after multiple administrations.

|  |  |  | Plasma SL: 1 ng/mL) | | |
| --- | --- | --- | --- | --- | --- |
| ID | Nation | Dosing | observed | Bayesian 9, 24, 48h | Bayesian 9, 24, 72h |
| 11 | Japan | 1.1 mg/kg q 24 h 5 days | 71.9 | 66.7 | 76.2 |
| 12 | Japan | 1.1 mg/kg q 24 h 5 days | 67.1 | 74.7 | 66.5 |
| 13 | Japan | 1.1 mg/kg q 24 h 5 days | 89.1 | 100.9 | 98.5 |
| 14 | Japan | 1.1 mg/kg q 24 h 5 days | 141.5 | 127.1 | 165.0 |
| 15 | Japan | 1.1 mg/kg q 24 h 5 days | 83.8 | 84.7 | 90.9 |
| 16 | Japan | 1.1 mg/kg q 24 h 5 days | 132 | 96.6 | 144.8 |
| 17 | Japan | 1.1 mg/kg q 24 h 5 days | 135.8 | 140.1 | 116.8 |
| 18 | Japan | 1.1 mg/kg q 24 h 5 days | 74.1 | 70.1 | 78.2 |
| 19 | Japan | 1.1 mg/kg q 24 h 5 days | 88 | 120.9 | 107.9 |
| 20 | Japan | 1.1 mg/kg q 24 h 5 days | 166.9 | 111.9 | 164.3 |
